# A universal N_2_O_4_-cavity strategy for precisely spaced, durable dual-atom ORR catalysts

**DOI:** 10.1039/d5sc07897k

**Published:** 2025-10-31

**Authors:** Guangxu Yao, Huijuan Zhang, Yangjun Luo, Chuanzhen Feng, Yu Wang

**Affiliations:** a State Key Laboratory of Power Transmission Equipment Technology, School of Chemistry and Chemical Engineering, Chongqing University Chongqing 400044 P. R. China wangy@cqu.edu.cn zhanghj@cqu.edu.cn; b BYD Automobile Industry Co. Ltd. Shenzhen Guangdong Province 518118 PR China

## Abstract

Diatomic catalysts offer greater promise than single-atom catalysts for the oxygen reduction reaction (ORR), yet simultaneously controlling atomic spacing, maximizing performance, and suppressing degradation remains challenging in conventional M–N_4_ frameworks. Here, we introduce an asymmetric N_2_O_4_ coordination in place of traditional N_4_ symmetry and, *via* an oxygen-coordinated polydentate ligand-electrospinning strategy, synthesize a MnMn-ON/C diatomic catalyst. MnMn-ON/C delivers a high half-wave potential *E*_1/2_ of 0.925 V, exceeding most reported asymmetric Mn-based diatomic catalysts. After 50 000 cycles it lost only 11 mV, and it operated stably in a zinc–air battery for 1000 hours without significant performance degradation, representing the best overall performance among asymmetric manganese-based diatomic systems. *In situ* spectroscopy combined with DFT reveals that N_2_O_4_-induced electronic coupling at Mn diatomic sites strengthens *OOH adsorption, while oxygen coordination tunes the density of states near the Fermi level. The cavity-confined architecture and strong Mn–O bonding jointly suppress migration and aggregation of active sites, enabling reversible valence regulation and recoverable activity. This asymmetric coordination-cavity strategy affords precise control of diatomic spacing and extends to other transition metals (Co, Cu, Ni), offering a general route to mitigate performance degradation in non-precious metal ORR catalysts.

## Abstract 2

MnMn-ON/C dual-atom catalyst *via* electrospinning with an oxygenated polydentate ligand. Features: asymmetric N_2_O_4_ pocket, oxygen donors improving ORR and durability, transferable to Co/Cu/Ni.

## Graphical abstract

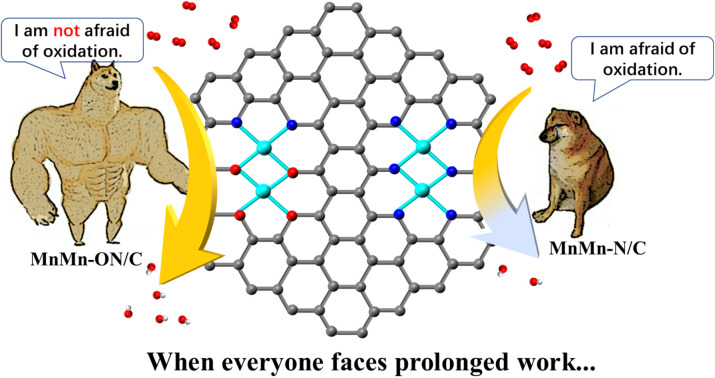

## Introduction

The escalating global energy and environmental pressures underscore the urgent need for sustainable, high-efficiency energy conversion technologies.^1–3^ The oxygen reduction reaction (ORR), the pivotal electrochemical step in fuel cells and metal–air batteries, ultimately governs the performance and commercial prospects of these devices.^4,5^ Yet their broad adoption is hampered by reliance on platinum catalysts. Advancing high-performance, low-cost, non-precious metal ORR catalysts is therefore a central scientific and technological priority for the clean-energy transition. Single-atom catalysts (SACs) have drawn intense interest for their fully exposed active sites and 100% atomic utilization.^6,7^ However, isolated sites enforce fixed adsorption modes, producing correlated adsorption energies across multi-step pathways.^8,9^ This scaling behavior hampers simultaneous optimization of adsorption and desorption for all intermediates, limiting overall activity. Dual-atom catalysts (DACs) offer a path to relax these constraints by enabling synergistic interactions and electron transfer between adjacent metals. They provide additional, cooperative adsorption motifs for multi-electron intermediates while tuning binding strengths.^10,11^ Most reported DACs, however, adopt symmetric coordination—typically M_2_N_6_ frameworks.^12,13^ Although such designs achieve dual-site synergy, each metal center remains in a symmetric N environment. The resultant symmetric charge distribution weakens adsorption of key ORR intermediates and slows reaction kinetics,^14^ representing a critical bottleneck to further progress in DAC development.^15^

Developing high-performance dual-atom catalysts faces several foundational challenges.^16,17^ First, precise, atomic-level control over the spacing between two metal atoms lacks robust, generalizable design rules.^18^ Current approaches largely depend on empirical screening, while conventional pyrolysis and impregnation often lead to aggregation and broad spacing distributions.^19^ For example, although some team's pyrolysis route yielded Mn dual-atom catalysts, the concurrent presence of clusters indicates room to further optimize ORR performance.^20^ Likewise, some team demonstrated the feasibility of dual-atom synthesis but focused mainly on single-element systems, leaving scope for more systematic investigation across metals and structures.^21^ Second, heteroatom coordination can boost ORR activity but introduces serious stability risks. Recent efforts to break the electronic and geometric symmetry of M–N_4_ sites—by varying the coordinating atoms and local environments (such as C, P, S, Cl)—have delivered notable performance gains, yet durability remains problematic. Electron enrichment at the active centers enhances intermediate activation but weakens the anchoring of metal sites. Bonds such as M–C,^22,23^ M–S,^24–27^ M–P,^28–30^ and M–Cl^31^ are prone to dissolution and reconstruction under electrochemical conditions, undermining long-term stability.^32^ For instance, some team's Fe single-atom catalyst achieved strong ORR activity by tuning Fe charge density to optimize adsorption of *OH, *O, and *OOH, but further improvements in durability are still needed.^29^ Finally, stability-protection strategies for Mn-based catalysts are especially limited. Under operating conditions, Mn can undergo complex, potential-dependent changes in valence, coordination, and interfacial behavior, complicating efforts to design targeted stabilization schemes.^6^ Existing studies have not fully clarified the intrinsic mechanisms of Mn active-site degradation, which in turn hinders the translation of these catalysts from laboratory demonstrations to practical applications.

To address the central challenges of dual-atom catalysts, we establish a comprehensive, multi-dimensional research framework. First, we synthesize MnMn-ON/C with asymmetric N_2_O_4_ coordination. Incorporating O atoms breaks symmetry and tunes the electronic structure of Mn active centers, enabling a half-wave potential of 0.925 V. At the same time, Mn–O coordination enhances durability during extended operation, with only an 11 mV loss in *E*_1/2_ after 50 000 CV cycles—mitigating the typical trade-off between activity and stability. Second, we integrate electrospinning with oxygen-coordinated polydentate ligands to achieve atomic-scale control over dual-atom spacing. This approach suppresses cluster formation and addresses the long-standing issue of uneven site separation in conventional syntheses. The strategy is general and extends to other transition metals (Co, Cu, Ni). Third, we comparatively probe degradation pathways in Mn-based catalysts. Conventional N-coordinated systems suffer cumulative activity loss due to Mn oxidation and Mn–N bond cleavage, which leads to Mn dissolution. By contrast, MnMn-ON/C primarily undergoes a transient increase in Mn valence under oxygen-rich conditions, causing reversible intrinsic activity decreases rather than bond rupture. This behavior makes post-operation recovery feasible by alleviating high oxidation states. Finally, *in situ* spectroscopy verifies the formation of ORR intermediates, while DFT calculations show that O coordination optimizes the Mn d-orbital configuration, improves catalytic performance, and reduces the propensity for further metal oxidation. Together, these advances provide a solid foundation for asymmetric dual-atom catalyst design and for suppressing performance degradation in non-precious metal coordination systems.

## Results and discussion

### Synthesis and characterization of MnMn-ON/C with demonstration of method generality

**Fig. 1:**
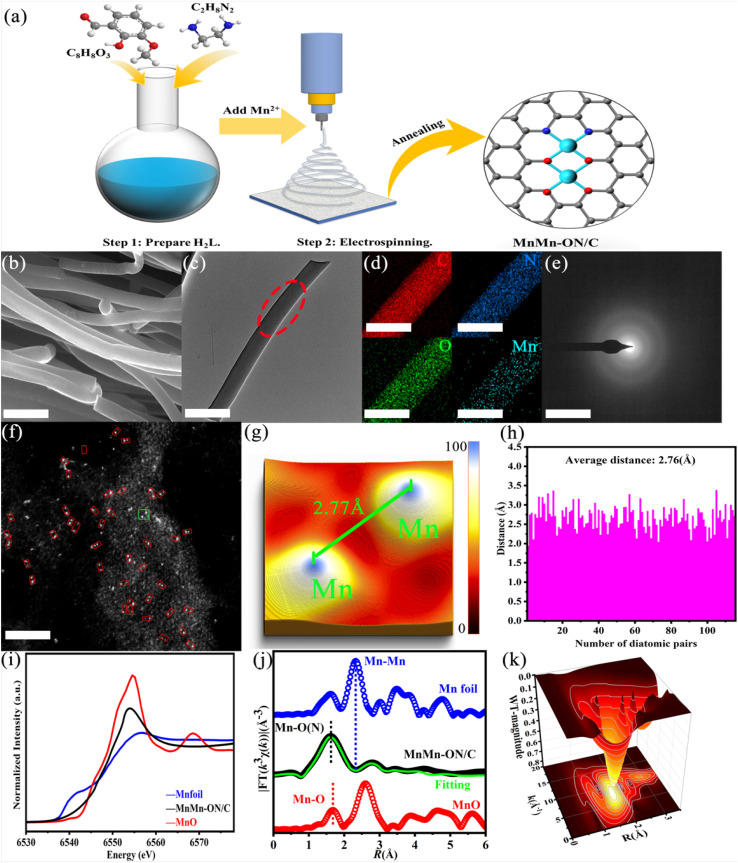
Synthetic strategy and structural characterization of MnMn-ON/C. (a) Schematic of the MnMn-ON/C fabrication process. (b) SEM micrograph (1 μm). (c) TEM micrograph (1 μm). (d) Elemental mapping of the region highlighted by the red dashed box in panel (c) (500 nm). (e) SAED pattern (5 nm^−1^). (f) AC-HAADF-STEM image (2 nm). (g) 3D structural model of Mn–Mn diatomic sites corresponding to the green box in panel (f). (h) Statistical distribution of Mn–Mn interatomic distances. (i) Mn K-edge XANES spectra. (j) Fourier-transformed EXAFS of selected samples, the green solid curve is the fitted Mn spectrum for MnMn-ON/C. (k) Wavelet transform contour of the Mn EXAFS.

To create highly dispersed and stable dual-atom catalytic sites, we developed a coordination-guided, ligand-templated synthesis (Fig. 1a). The process has two stages: (1) prepare a multidentate oxygen-containing organic ligand (H_2_L), coordinate it with metal ions to form a well-defined metal–ligand precursor (Mn complex). (2) Blend the precursor with polymers, form fibrous intermediates by electrospinning, and then anneal to construct Mn–O–Mn bridged dual-atom sites on a carbon support (MnMn-ON/C). By substituting the metal salts, this strategy generalizes to Co, Ni, and Cu dual-atom catalysts (Fig. S1).

The ligand H_2_L was obtained by a Schiff-base condensation of C_8_H_8_O_3_ with ethylenediamine in ethanol at 50–55 °C, followed by recrystallization to afford H_2_L in 69% yield (C_18_H_20_N_2_O_4_). ^1^H NMR (Fig. S2a) and mass spectrometry (Fig. S2b) confirmed the expected structure and purity, providing a reliable precursor for coordination and templating. We then elucidated the coordination stoichiometry and binding mode with Mn^2+^ using colorimetric observation, ESI-MS, FT-IR, and fluorescence titration. Upon adding 2 equivalents of Mn^2+^ to a solution of H_2_L (1 equivalent), the solution color changed immediately, indicating complex formation, ESI-MS showed a peak at *m*/*z* = 436.06 (Fig. S2c), consistent with the complex. FT-IR (Fig. S2d) revealed the disappearance of the O–H stretch at ∼3443 cm^−1^ (deprotonated phenolates engaged in coordination), a slight red shift of C=N from ∼1615 to ∼1610 cm^−1^ (nitrogen coordination affecting conjugation), and a shift of the Ar–O stretch from ∼1283 to ∼1247 cm^−1^ (aromatic oxygen coordination). Fluorescence titration (H_2_L 1 × 10^−5^ mol L^−1^, *λ*_ex_ = 280 nm, Mn^2+^ 1 × 10^−3^ mol L^−1^) showed strong ligand emission at ∼380 nm that quenched progressively and plateaued near 2 equivalents of Mn^2+^ (Fig. S2e), break-point analysis across repeats gave a clear inflection at Mn^2+^/H_2_L = 2.0 (Fig. S2f), establishing a 1 : 2 H_2_L : Mn^2+^ stoichiometry. Time-dependent fluorescence and UV visualization (Fig. S2g) indicated good complex stability. Together, these data confirm a multidentate 1 : 2 coordination that molecularly templates subsequent dual-atom site construction on carbon.

Building on this precursor, we co-blended the Mn complex with PVP and PAN and produced uniform fibers by electrospinning, precisely controlling diameter *via* voltage, tip-to-collector distance, flow rate, and ambient humidity. The as-spun fibers were highly flexible (Fig. S3a). After annealing at 600 °C, the carbonized mats retained appreciable flexibility (Fig. S3b), indicating good mechanical integrity suitable for flexible devices (*e.g.*, flexible Zn–air batteries). Characterization confirms that combining ligand templating with electrospinning and controlled annealing yields well-dispersed, architected Mn–O–N bridged dual-atom sites on carbon fibers. Replacing the metal salts under the same workflow affords CoCo-ON/C, NiNi-ON/C, and CuCu-ON/C (Fig. S1), demonstrating the generality of this ligand-templated approach across multiple transition metals.

SEM imaging (Fig. S4a) reveals continuous MnMn-ON/C fibers with diameters predominantly in the 300–400 nm range (Fig. S4b). Higher-magnification SEM (Fig. 1b) provides additional surface and morphological detail, confirming uniform fiber formation. TEM images (Fig. 1c) show straight fibers with smooth surfaces, while EDS elemental maps (Fig. 1d) demonstrate a homogeneous distribution of Mn, N, O, and C across the fibers. SAED patterns (Fig. 1e) lack polycrystalline rings, indicating an overall amorphous carbon matrix and the absence of metallic Mn phases, consistent with atomically dispersed Mn. Atomic-resolution HAADF-STEM further assesses Mn dispersion. As shown in Fig. 1f, Mn occurs predominantly as closely spaced atom pairs (highlighted in red) uniformly distributed on the carbon support, occasional single Mn atoms are present, and no metal clusters are detected. A representative Mn pair (green box) was used to construct a 3D structural model (Fig. 1g), in which C, N, and O appear darker due to weaker scattering, while Mn appears bright blue. The measured Mn–Mn distance is 2.77 Å. Population statistics from multiple images (Fig. 1h) show Mn–Mn separations mainly between 2.0 and 3.2 Å, with an average of 2.76 Å. These observations support a dual-atom configuration formed from a single H_2_L ligand coordinating two Mn^2+^ ions, where the N_2_O_2_ and O_4_ coordination cavities spatially confine the interatomic distance.

X-ray absorption spectroscopy (XAS) was used to resolve the Mn coordination environment and oxidation state. The Mn K-edge XANES (Fig. 1i) shows clear distinctions among Mn foil, MnMn-ON/C, and MnO. The absorption edge of MnMn-ON/C falls between Mn foil and MnO, indicating an average Mn valence between 0 and +2. *k*^3^-weighted EXAFS of the Fourier transform (Fig. 1j) further clarifies the local bonding environment. In MnMn-ON/C, the Mn–N and Mn–O bond lengths are approximately 1.77 Å and 1.96 Å, respectively. The characteristic Mn–Mn peak of metallic Mn at 2.45 Å is absent, ruling out appreciable Mn metal clusters. Combined with electron microscopy, the weak signal at 2.78 Å can be assigned to neighboring Mn atoms, indicating a Mn–Mn distance of roughly 2.78 Å. Wavelet transform analyses (Fig. 1k and S5a, b) are consistent with the *R*-space assignments, and fitting in *k*-space also shows good agreement (Fig. S5c–e), the fitted parameters are summarized in Table S1. Overall, XAS and AC-HAADF-STEM together corroborate the presence of stable Mn dual-atom pairs in MnMn-ON/C. To isolate the role of oxygen coordination, a control catalyst, MnMn-N/C, containing only N coordination, was synthesized. SEM (Fig. S6a) shows straight, unbranched fibers, HAADF-STEM (Fig. S6b) reveals smooth surfaces, and EDS maps (Fig. S6c) confirm uniform elemental dispersion. SAED (Fig. S6d) shows no diffraction spots, indicating the absence of Mn particles or clusters, analogous to MnMn-ON/C. Conventional analyses further compare the two catalysts.

XRD (Fig. S7a) displays a single broad reflection near 24° for both samples, assignable to graphitic (002), with no peaks for metallic Mn, consistent with atomic-level dispersion. Nitrogen sorption (Fig. S7b) shows type-IV isotherms and similar surface areas—232.13 m^2^ g^−1^ for MnMn-ON/C and 225.72 m^2^ g^−1^ for MnMn-N/C—indicating comparable porosity and morphology. These results ensure that differences in catalytic behavior can be attributed primarily to the distinct coordination environments rather than to textural factors. Raman spectroscopy (Fig. S7c) shows two characteristic bands at 1346 cm^−1^ (D) and 1580 cm^−1^ (G) for both catalysts. The intensity ratio (*I*_D_/*I*_G_) is 1.2 for MnMn-ON/C and 1.1 for MnMn-N/C, indicating a higher defect density in MnMn-ON/C (*e.g.*, edges, heteroatom sites, increased sp^3^ content, or smaller graphitic domains). Such defects can facilitate charge transport and benefit ORR kinetics. XPS survey spectra are broadly similar (Fig. S7d). MnMn-ON/C exhibits a slightly stronger O 1s signal at 532 eV, consistent with greater oxygen coordination. High-resolution Mn 2p spectra show MnMn-N/C with Mn 2p_1/2_ at 654.0 eV and Mn 2p_3/2_ at 642.0 eV (Fig. S7e).^33,34^ MnMn-ON/C displays a small positive shift in both peaks, attributable to the higher electronegativity of O relative to N: oxygen withdraws more electron density from Mn, lowering the local electron cloud and increasing core-level binding energies. Consistently, the Mn K-edge XANES of MnMn-N/C (Fig. S7f) positions its absorption between Mn foil and MnMn-ON/C, aligning with the XPS trend. Together, XANES and XPS confirm that the coordination environment significantly tunes the Mn electronic structure. Relative to N coordination, O coordination in MnMn-ON/C reduces Mn electron density and raises the effective oxidation state, a factor expected to influence catalytic behavior. FT EXAFS of MnMn-N/C (Fig. S7g) resolves its local coordination, the fit (red line) shows good agreement in both *R*-space and *k*-space (Fig. S7h), with detailed parameters summarized in Table S1. These results provide a consistent structural and electronic baseline for comparing MnMn-ON/C and MnMn-N/C.

**Fig. 2:**
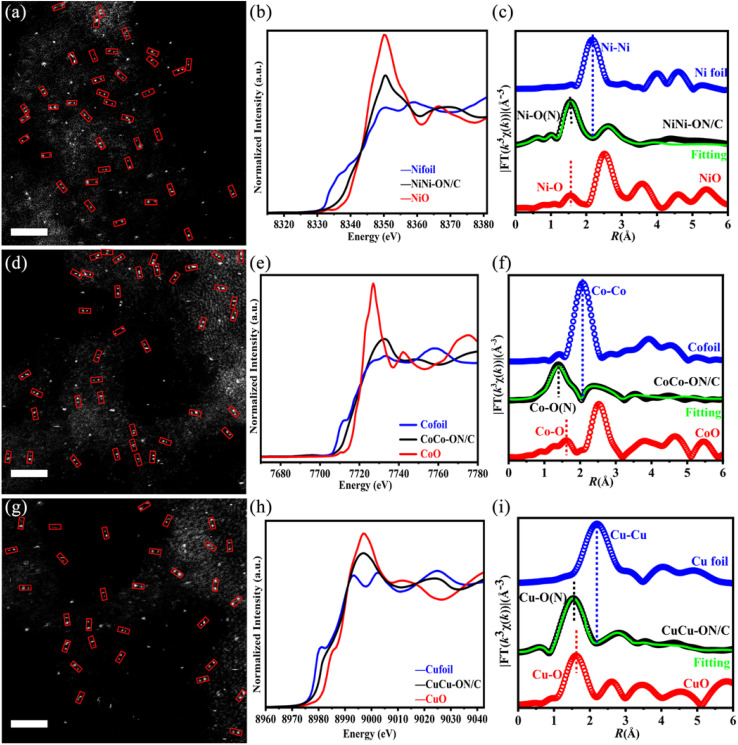
Generalizability of the synthesis strategy across Ni, Co, and Cu systems. (a) AC-HAADF-STEM image of the NiNi-ON/C catalyst (2 nm). (b) Ni K-edge XANES spectra. (c) Fourier-transformed EXAFS of NiNi-ON/C, Ni foil, and NiO, the green solid curve denotes the fitted Ni spectrum for NiNi-ON/C. (d) AC-HAADF-STEM image of the CoCo-ON/C catalyst (2 nm). (e) Co K-edge XANES spectra. (f) Fourier-transformed EXAFS of CoCo-ON/C, Co foil, and CoO, the green solid curve denotes the fitted Co spectrum for CoCo-ON/C. (g) AC-HAADF-STEM image of the CuCu-ON/C catalyst (2 nm). (h) Cu K-edge XANES spectra. (i) Fourier-transformed EXAFS of CuCu-ON/C, Cu foil, and CuO, the green solid curve denotes the fitted Cu spectrum for CuCu-ON/C.

The oxygen-containing polydentate ligand plus electrospinning approach offers four advantages: (1) High atomic utilization: each ligand anchors two metal atoms, minimizing metal loss. (2) Precise spatial control: the rigid ligand scaffold dictates metal–metal spacing with accuracy. (3) Mild processing: a 600 °C anneal circumvents energy-intensive atom-trapping procedures. (4) Broad applicability: the method extends beyond Mn to multiple transition metals. Following the same protocol, NiNi-ON/C, CoCo-ON/C, and CuCu-ON/C were prepared. Their XRD patterns (Fig. S8a) show only a prominent reflection near 24° assigned to graphitic (002), with no metal or alloy peaks, indicating atomic-level dispersion. SEM images (Fig. S8b–d) confirm morphologies comparable to MnMn-ON/C. AC-HAADF-STEM and XAS establish dual-atom dispersion for Ni, Co, and Cu on carbon (Fig. 2a, d and g), with interatomic distance statistics summarized in Fig. S9a–c. K-edge XANES places all three metals at oxidation states between 0 and +2 (Fig. 2b, e and h). *R*-space EXAFS fits identify dominant M–O and M–N coordination (Fig. 2c, f and i). Wavelet transforms of k^3^-weighted EXAFS (Fig. S9d–l) and corresponding *k*-space fits (Fig. S10) are consistent with these assignments. Detailed fitting parameters are provided in Table S1. Together, these results verify that the ligand-templated electrospinning strategy is general and reproducible across Mn, Ni, Co, and Cu, while preserving dual-atom architectures and controlled local coordination.

### Electrochemical evaluation of MnMn-ON/C for the ORR

**Fig. 3:**
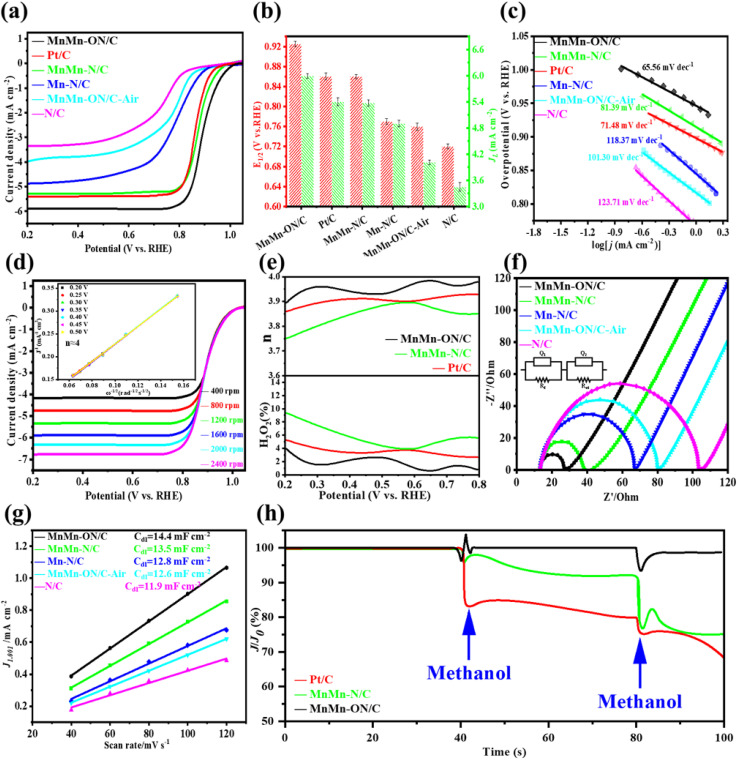
Electrochemical evaluation of the catalysts. (a) LSV profiles for the different catalysts. (b) Half-wave potentials and limiting current densities extracted from the LSV data. (c) Tafel slopes derived from the LSV measurements. (d) Relationship between current density and rotation rate for catalyst MnMn-ON/C (inset shows the calculated electron transfer number). (e) H_2_O_2_ output for three representative catalysts. (f) EIS Nyquist plots for the catalyst set. (g) Double-layer capacitive current as a function of scan rate. (h) Methanol tolerance tests for the three catalysts.

Electrochemical ORR measurements were carried out in 0.1 M KOH. Because fiber diameter affects microstructure and mass transport, MnMn-ON/C samples with different diameters were screened by LSV. The best half-wave potential was obtained for 300–400 nm fibers (Fig. S11), thinner or thicker fibers lowered activity by hindering electron transport, site accessibility, and diffusion. All subsequent samples were therefore prepared under identical electrospinning conditions to ensure comparability. Under the optimized conditions, MnMn-ON/C, MnMn-N/C, Mn-N/C, MnMn-ON/C-Air, N/C, and commercial Pt/C were benchmarked. LSV curves (Fig. 3a) show MnMn-ON/C with the most positive onset potential and the highest limiting current, while N/C is the least active. Statistical analysis over repeated tests (Fig. 3b) gives MnMn-ON/C an *E*_1/2_ of 0.925 V and *J*_L_ of 5.98 mA cm^−2^, outperforming Pt/C (*E*_1/2_ = 0.860 V, *J*_L_ = 5.41 mA cm^−2^) and meeting the U.S. DOE target ≥ 0.90 V. MnMn-N/C is comparable to Pt/C, whereas Mn-N/C reaches only 0.771 V, underscoring the advantage of dual-atom sites over single atoms. The gap between MnMn-ON/C and Mn-N/C (Δ*E*_1/2_ = 154 mV) highlights electronic coupling and spatial synergy in the dual-atom architecture. MnMn-ON/C-Air (0.762 V, 4.01 mA cm^−2^) and N/C (0.721 V, 3.44 mA cm cm^−2^) show substantially lower activity. Tafel analysis (Fig. 3c) further clarifies kinetics: MnMn-ON/C exhibits a slope of 65.56 mV dec^−1^, better than Pt/C (71.48 mV dec^−1^), indicating lower kinetic barriers and faster ORR dynamics. RRDE measurements assess selectivity and pathways. MnMn-ON/C exhibits an electron transfer number of 3.93 (Fig. 3d) and an H_2_O_2_ yield of only 3% (Fig. 3e), both of which are better than those of the other samples under identical conditions (Fig. S12). These results demonstrate that MnMn-ON/C predominantly follows an efficient 4-electron pathway while suppressing H_2_O_2_ formation, a key advantage for fuel cells and Zn–air batteries.

Electrochemical impedance spectroscopy (EIS, Fig. 3f) was used to probe charge-transfer behavior. MnMn-ON/C shows the smallest charge-transfer resistance *R*_ct_ = 15.21 Ω, substantially lower than MnMn-N/C (26.21 Ω), Mn-ON/C (55.52 Ω), MnMn-ON/C-Air (68.23 Ω), and N/C (99.52 Ω). This underscores the advantages of dual-atom architectures: delocalization between adjacent Mn atoms improves conductivity, while mixed O/N coordination tunes the metal centers to facilitate rapid electron transfer and accelerate ORR. Double-layer capacitance *C*_dl_, extracted from cyclic voltammetry in the non-faradaic region (Fig. S13), was used as a proxy for electrochemically accessible surface area. MnMn-ON/C delivers the highest *C*_dl_ (14.4 mF cm^−2^, Fig. 3g), indicating a larger accessible active surface, consistent with its superior ORR activity. To decouple geometric effects and ensure fair comparison (Fig. S14), LSV currents were normalized by ECSA and BET surface area, MnMn-ON/C remains more active than MnMn-N/C after normalization. Methanol tolerance was assessed to gauge resistance to fuel impurities (Fig. 3h). After a methanol injection at 40 s, MnMn-ON/C shows negligible current loss, whereas MnMn-N/C stabilizes at ∼93% and Pt/C exhibits clear decay. Following a second injection at 80 s, MnMn-ON/C maintains ∼96% of its current, while MnMn-N/C and Pt/C drop sharply. These results demonstrate strong anti-poisoning behavior for MnMn-ON/C, supporting its suitability for fuel-cell applications. Compared with leading literature catalysts (Table S2), MnMn-ON/C achieves top-tier ORR metrics across multiple benchmarks, highlighting the promise of dual-atom designs for practical energy devices.

### Performance evaluation of flexible and liquid Zn–air batteries

**Fig. 4:**
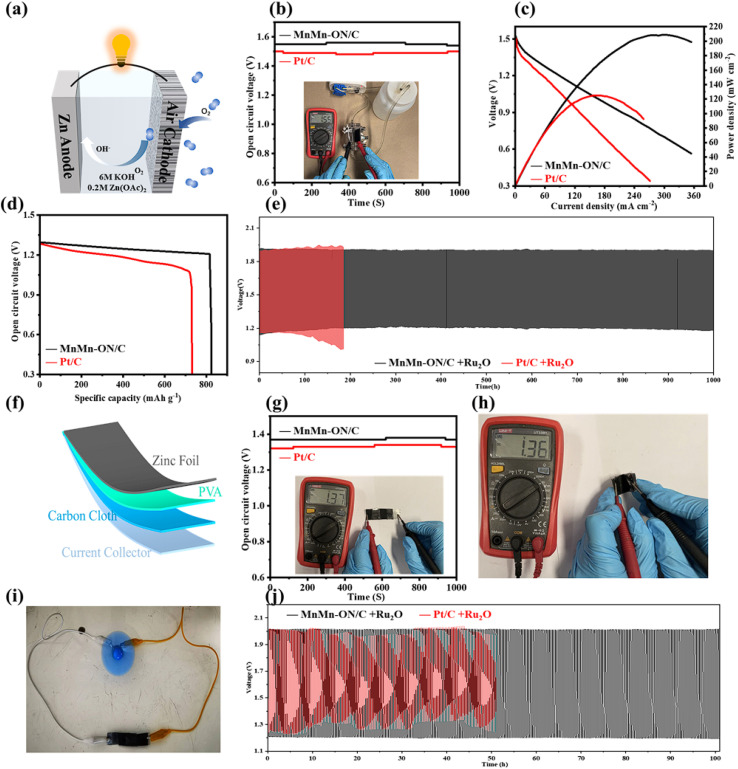
Performance of MnMn-ON/C in liquid and flexible Zn–air batteries. (a) Schematic of the liquid Zn–air cell configuration. (b) Open-circuit voltage comparison among Zn–air cells. (c) Discharge polarization curves and corresponding power density comparisons. (d) Specific capacity comparison of Zn–air cells. (e) Galvanostatic charge–discharge profiles at 5 mA cm^−2^. (f) Schematic of the flexible Zn–air battery. (g) Open-circuit voltage comparison for flexible cells. (h) Open-circuit voltage measured after bending. (i) Photograph of a flexible Zn–air battery powering a miniature fan. (j) Galvanostatic charge–discharge profiles at 2 mA cm^−2^.

Given its strong ORR activity and methanol tolerance, MnMn-ON/C was deployed as the air cathode in rechargeable liquid zinc–air batteries (ZABs) for practical evaluation (Fig. 4a). The cells used MnMn-ON/C as the cathode, zinc foil as the anode, and KOH electrolyte. The open-circuit voltage reached 1.55 V, surpassing Pt/C-based ZABs (1.50 V, Fig. 4b). Power density was derived from discharge profiles at varied current densities (Fig. 4c). MnMn-ON/C ZABs delivered a peak power density of 209.1 mW cm^−2^, 61% higher than Pt/C + RuO_2_ (129.7 mW cm^−2^). The advantage stems from higher voltage retention under load, reflecting favorable ORR kinetics and mass transport. At 5 mA cm^−2^, MnMn-ON/C achieved a specific discharge capacity of 817.7 mA h g_Zn_^−1^, outperforming Pt/C (735.2 mA h g_Zn_^−1^, Fig. 4d), indicating efficient Zn utilization *via* a dominant four-electron pathway with minimal side reactions. For long-term cycling, a bifunctional MnMn-ON/C + RuO_2_ cathode enabled stable operation over 1000 h with negligible voltage decay, whereas Pt/C + RuO_2_ failed within 150 h (Fig. 4e). Discharge curves across current densities (Fig. S15a) showed stable voltage output, demonstrating adaptability to diverse power demands.

Building on the membrane flexibility of MnMn-ON/C, foldable solid-state ZABs were assembled (Fig. 4f). The open-circuit voltage reached 1.37 V, higher than Pt/C (1.32 V, Fig. 4g). Under bending, the voltage decreased by only 0.01 V to 1.36 V (Fig. 4h), confirming mechanical and electrochemical stability. The flexible MnMn-ON/C ZAB achieved a maximum power density of 81.29 mW cm^−2^, outperforming Pt/C (52.47 mW cm^−2^, Fig. S15b), indicating sustained catalytic performance in solid-state configurations. A single flexible cell powered a miniature fan (Fig. 4i), illustrating potential for portable electronics. In cycling tests, MnMn-ON/C + RuO_2_ maintained stable operation for 100 h, while Pt/C + RuO_2_ degraded within 50 h (Fig. 4j). Charge–discharge curves at different bending angles (Fig. S15c) further verified mechanical robustness for wearable applications. Across both liquid and flexible solid-state ZABs, MnMn-ON/C demonstrated superior electrochemical performance and stability. Relative to literature benchmarks (Table S3), it delivers leading metrics, underscoring the promise of dual-atom catalysts for next-generation energy storage.

### Performance fade in ORR catalysts: causes, diagnostics, and solutions

In ORR catalysis, single-atom catalysts typically degrade *via* two pathways: structural and electronic. Structural degradation involves breaking metal–support coordination bonds and the physical loss of active metal sites, it is irreversible. Electronic degradation arises from increased metal oxidation state and changes in electronic structure during operation, it lowers intrinsic activity without breaking bonds and is, in principle, reversible. To probe these mechanisms and evaluate recovery strategies, accelerated durability tests (ADT) were performed using cyclic voltammetry (CV).

Commercial Pt/C showed pronounced degradation under extended CV. After 10 000 cycles, its ORR performance (Pt/C-CV1W) dropped markedly, and after 50 000 cycles, Pt/C-CV5W degraded to near the activity of the N/C support, underscoring the stability limits of conventional precious-metal catalysts (Fig. S16).

**Fig. 5:**
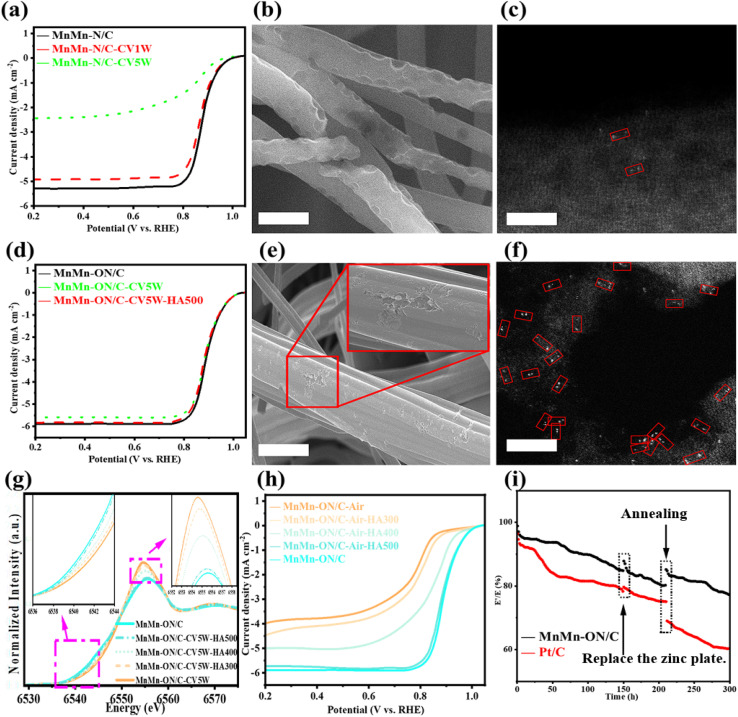
Strategies to mitigate ORR performance decay. (a) LSV profiles of MnMn-N/C, MnMn-N/C-CV1W, and MnMn-N/C-CV5W. (b) SEM micrograph of MnMn-N/C-CV5W (200 nm). (c) AC-HAADF-STEM image of MnMn-N/C-CV5W (2 nm). (d) LSV profiles of MnMn-ON/C, MnMn-ON/C-CV5W, and MnMn-ON/C-CV5W-HA500. (e) SEM micrograph of MnMn-ON/C-CV5W (200 nm), inset: magnified view of the red-boxed region. (f) AC-HAADF-STEM image of MnMn-ON/C-CV5W (2 nm). (g) Mn K-edge XANES for MnMn-ON/C, MnMn-ON/C-CV5W-HA500, -HA400, -HA300, and MnMn-ON/C-CV5W. (h) LSV profiles of MnMn-ON/C-Air, MnMn-ON/C-Air-HA500, -HA400, -HA300, and MnMn-ON/C. (i) Normalized discharge voltage of MnMn-ON/C and Pt/C during continuous operation in a Zn–air battery at 5 mA cm^−2^.

MnMn-N/C exhibited severe decay in ADT. After 10 000 cycles, the half-wave potential of MnMn-N/C-CV1W decreased by 25 mV relative to the pristine catalyst. Extending to 50 000 cycles further widened the performance gap (Fig. 5a). SEM revealed extensive surface corrosion in MnMn-N/C-CV1W, with numerous pores across the fiber surface (Fig. 5b). TEM showed deep pitting of the carbon fibers and compromised structural integrity (Fig. S17a). Corresponding elemental maps recorded a pronounced drop in local Mn signal (Fig. S17b). AC-HAADF-STEM confirmed a strong decrease in Mn atom-pair density, with only sporadic single Mn atoms remaining, evidencing dissolution and loss of active sites (Fig. 5c). ICP-MS corroborated this: Mn content declined from 4.23 wt% (fresh) to 3.59 wt% after 10 000 cycles (−15.1%), and to 2.03 wt% after 50 000 cycles (47.99% of the initial content, Table S4). These findings identify metal-site loss as the primary cause of performance decline in MnMn-N/C—an irreversible failure stemming from the thermodynamic instability of Mn–N coordination under prolonged high potentials, which promotes oxidation and detachment. Concurrent electrochemical corrosion of the carbon support further destabilizes the local coordination environment.

In sharp contrast to MnMn-N/C, MnMn-ON/C shows outstanding durability. After 50 000 CV cycles, its half-wave potential decreased by only 11 mV relative to the pristine sample (Fig. 5d). SEM revealed only minor surface defects (Fig. 5e), and TEM showed superficial damage that did not penetrate the fiber, with elemental distributions comparable to the pre-reaction state (Fig. S18). AC-HAADF-STEM confirmed that Mn atom pairs remained randomly dispersed on the carbon surface without a noticeable change in density (Fig. 5f). ICP analysis showed a small decrease in Mn content from 4.20 wt% to 3.91 wt% (−7%), far less than the loss observed for MnMn-N/C (Table S4). These results indicate that metal-site loss contributes only marginally to the modest activity decline. The superior stability of MnMn-ON/C is likely linked to two factors.^9,12,35,36^ First, the N_2_O_4_ cavity provides a confined coordination environment that limits metal migration and aggregation. Second, oxygen incorporation tunes the Mn electronic structure, stabilizing the d-electron configuration and lowering the tendency toward over-oxidation—an interpretation supported by subsequent DFT results. During ORR, Mn centers interact with O_2_ and OH^−^ and shift from relatively low to higher oxidation states. Given that Mn–N bonds have bond energies of approximately 200–250 kJ mol^−1^, whereas Mn–O bonds reach 350–400 kJ mol^−1^, the stronger Mn–O bonding offers enhanced thermodynamic stability.

Oxidation of the metal centers is therefore another key driver of performance decay. Building on this, suppressing high Mn oxidation states after prolonged operation can enable activity recovery. Annealing under a reducing atmosphere is effective for this purpose. Hydrogen acts as a mild reductant that can lower Mn oxidation state without over-reducing to metallic Mn, while argon dilutes H_2_ to control reduction extent and excludes reactive impurities (such as O_2_ and H_2_O). The temperature program is designed to balance reduction efficacy and structural integrity: 300 °C tests the feasibility of low-temperature reduction near the onset for many metal oxides. 400 °C provides greater thermodynamic driving force while limiting structural rearrangement. 500 °C approaches, but remains below, the original synthesis temperature (600 °C), promoting effective reduction while preserving the coordination cavity.

Under H_2_/Ar atmosphere, MnMn-ON/C-CV5W was treated at 300 °C, 400 °C, and 500 °C for 40 minutes, respectively. X-ray absorption near-edge structure (XANES) spectroscopy analysis of the Mn K-edge revealed that MnMn-ON/C-CV5W-HA500, treated at 500 °C, exhibited spectral intensity nearly identical to that of pristine MnMn-ON/C. Although MnMn-ON/C-CV5W-HA300 and MnMn-ON/C-CV5W-HA400 also showed intensity trends approaching that of MnMn-ON/C, the effect was less pronounced than that observed for MnMn-ON/C-CV5W-HA500 (Fig. 5g). Correspondingly, X-ray photoelectron spectroscopy (XPS) analysis demonstrated that the oxygen content in MnMn-ON/C-CV5W-HA500 was nearly consistent with that of pristine MnMn-ON/C (Fig. S19). Linear sweep voltammetry (LSV) test results were consistent with XANES analysis, showing that the half-wave potential of MnMn-ON/C-CV5W-HA500 was only 3 mV lower than that of MnMn-ON/C (Fig. 5d). ICP testing revealed that the Mn content in MnMn-ON/C-CV5W-HA500 was 3.89%, virtually identical to the 3.91% in MnMn-ON/C-CV5W, indicating that annealing treatment does not cause further detachment of Mn species. Based on these observations, it can be reasonably concluded that annealing at 500 °C for 40 minutes under H_2_/Ar atmosphere effectively mitigates the oxidation state of Mn in MnMn-ON/C-CV5W, reducing high-valence Mn to lower oxidation states approaching those in MnMn-ON/C, thereby achieving partial activity recovery.

To further validate the hypothesis of oxidation-induced performance degradation followed by reduction-induced performance recovery, two key experiments were conducted. Experiment 1: If the hypothesis is correct, then intentionally oxidized MnMn-ON/C samples should recover to performance levels approaching the original sample after H_2_/Ar reduction treatment. Therefore, air-annealed MnMn-ON/C-Air samples were used as “artificially oxidized” controls. MnMn-ON/C-Air was treated under H_2_/Ar atmosphere at 300 °C, 400 °C, and 500 °C for 40 minutes, respectively, and their ORR performance was subsequently evaluated (Fig. 5h). Results showed that the ORR electrocatalytic activities of MnMn-ON/C-Air-HA300, MnMn-ON/C-Air-HA400, and MnMn-ON/C-Air-HA500 exhibited an increasing trend, with MnMn-ON/C-Air-HA500 achieving half-wave potential and limiting current density slightly inferior to MnMn-ON/C. Considering that the preparation difference between MnMn-ON/C-Air and MnMn-ON/C lies only in the final annealing atmosphere, where the former may have undergone Mn oxidation by oxygen participation during annealing in air, these results demonstrate that mitigating high Mn oxidation states through H_2_/Ar atmosphere annealing can indeed restore catalyst activity.

Experiment 2: To validate the reduction-based recovery strategy under practical conditions, zinc–air battery testing was conducted for two reasons: *Real-world validation*: compared with three-electrode tests, zinc–air batteries better emulate operating environments, allowing assessment of practical efficacy. *Long-term stability*: continuous 200 h operation evaluates whether reduced catalysts maintain performance under sustained load. MnMn-ON/C and Pt/C were each assembled into zinc–air batteries and discharged at a constant 5 mA cm^−2^ for extended stability evaluation (Fig. 5i). At 150 h, the zinc anodes were replaced, both cells showed some recovery, likely due to renewal of the reaction interface. At 200 h, both cathodes were annealed at 500 °C in H_2_/Ar for 40 minutes, then reassembled for continued operation. The MnMn-ON/C cell increased its discharge platform from 80% of the initial value to 86%, indicating partial recovery of electrochemical activity. In contrast, the Pt/C cell suffered pronounced, irreversible voltage loss. These two experiments fully demonstrate the reliability and effectiveness of the annealing strategy.

To test whether activity recovery was a mere temperature effect rather than a true reduction process, control anneals were performed in pure Ar at the same temperatures. If temperature alone were responsible, pure Ar treatment should yield similar outcomes as H_2_/Ar. Instead, conventional Ar annealing failed to mitigate Mn oxidation. After treating MnMn-ON/C-CV5W at 300 °C, 400 °C, and 500 °C for 40 minutes, Mn K-edge XANES showed virtually no change (Fig. S20a). Even at 600 °C for 40 minutes, only a slight decrease in apparent oxidation state was observed, while EXAFS Fourier-transformed *k*^3^-weighted *χ*(*k*) revealed a clear Mn–Mn scattering path at *R* = 2.45 Å, indicating metal aggregation and possible cluster formation (Fig. S20b). Given that XAS reflects average valence, the slight drop is attributable to low-valence Mn within clusters lowering the overall signal. AC-HAADF-STEM directly confirmed Mn clusters in MnMn-ON/C-CV5W-A600 (Fig. S20c). These findings show that pure Ar annealing neither effectively reduces Mn oxidation states nor preserves atomic dispersion at elevated temperatures. These results clarify the degradation and recovery landscape. Unlike the irreversible rupture of Mn–N coordination, Mn oxidation within the Mn–O coordination framework primarily manifests as changes in oxidation state rather than bond cleavage, meeting the thermodynamic criteria for reversibility. Moreover, the coordination cavity confines Mn atom pairs, lowering the diffusion activation energy and enabling reduction under relatively mild conditions, thus avoiding high-temperature restructuring that drives clustering. Together, these factors explain why MnMn-ON/C is not only more stable than MnMn-N/C but also amenable to low-temperature reduction-based recovery.

To assess generality, universality tests were designed under strict variable control: preparation procedures were kept identical while only the metal species (Ni/Co/Cu) was varied. Comparing stability across different metals within the same cavity environment validated the stabilization mechanism. Both current–time profiles (Fig. S21) and ICP measurements (Table S5) showed excellent stability for these catalysts, confirming that the robustness of the N_2_O_4_ cavity is universal and not limited to Mn.

### ORR reaction mechanism on the MnMn-ON/C catalyst

**Fig. 6:**
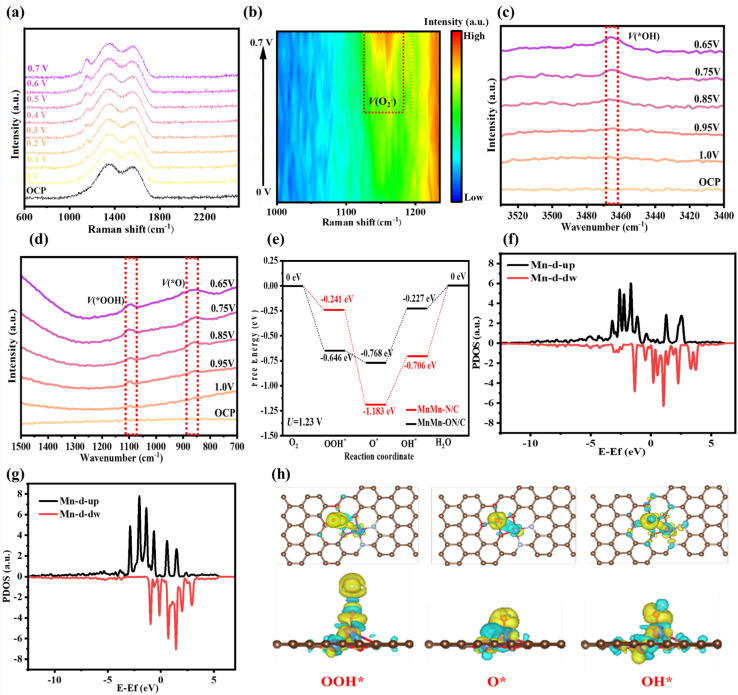
Mechanistic insights into ORR on the MnMn-ON/C catalyst. (a) *In situ* Raman spectra of MnMn-ON/C collected at different applied potentials in O_2_-saturated 0.1 M KOH. (b) Enlarged *in situ* Raman contour map focusing on the 1000–1250 cm^−1^ region. (c and d) *In situ* ATR-SEIRAS measurements of MnMn-ON/C during ORR in 0.1 M KOH. (e) Gibbs free-energy profiles for the four-electron ORR pathway on MnMn-ON/C *versus* MnMn-N/C. (f) Projected density of states (PDOS) for Mn in MnMn-N/C. (g) Projected density of states (PDOS) for Mn in MnMn-ON/C. (h) Top and side views of 3D differential charge-density distributions for three key ORR intermediates on MnMn-ON/C.

To uncover why MnMn-ON/C delivers outstanding ORR activity, we combined advanced characterization with density functional theory (DFT) to interrogate its reaction mechanism. *In situ* Raman spectroscopy, which tracks surface intermediates during operation, provided direct evidence for oxygenated species forming on MnMn-ON/C (Fig. 6a). At 0.5 V, a characteristic band near 1150 cm^−1^, assigned to the O–O stretch of O_2_^−^, appeared, indicating activation of O_2_ and formation adsorbed intermediates.^35,37,38^ Raising the potential to 0.7 V markedly increased the peak intensity, consistent with a higher surface coverage of oxygenated intermediates. This evolution is clearly visualized in the 1000–1250 cm^−1^ contour plots (Fig. 6b), which map the potential-dependent build-up of intermediates during ORR. To pinpoint the key species along the pathway, we further conducted *in situ* ATR-SEIRAS for real-time monitoring on MnMn-ON/C. Distinct features at 3470 cm^−1^, 1100 cm^−1^, and 860 cm^−1^ were observed and assigned based on prior reports and theoretical expectations to *OH, *OOH, and *O, respectively (Fig. 6c).^39–42^ As the applied potential was lowered, the absorption signals of all three intermediates increased significantly (Fig. 6d), evidencing their progressive formation and accumulation under working conditions. Together, these observations substantiate a four-electron ORR pathway on MnMn-ON/C and provide direct experimental support for the proposed reaction sequence that DFT subsequently rationalizes.

DFT-based reaction free-energy diagrams clarify the intrinsic catalytic advantages of MnMn-ON/C (Fig. 6e). Relative to a conventional N coordination, the mixed N_2_O_4_ environment strengthens adsorption of the first-step *OOH intermediate, facilitating initial O_2_ activation and lowering the entry barrier for ORR. At the standard potential of 1.23 V, Mn sites within N_2_O_4_ coordination display reduced barriers at the rate-determining step, primarily because *OH adsorption is moderately weakened. This prevents overly strong binding during product desorption and achieves a more balanced adsorption strength across intermediates. Top/side-view geometries for MnMn-N/C and MnMn-ON/C through the three intermediate steps visualize these structural changes (Fig. S22 and S23). Electronic-structure analysis *via* PDOS reveals how O coordination tunes Mn centers (Fig. 6f and g). Incorporating O into the coordination sphere shifts spin-up and spin-down states upward below the Fermi level and increases the density of states near the Fermi level. This redistribution optimizes the Mn d-electron configuration, stabilizes electron distribution, and suppresses further oxidation of the metal centers, enhancing both stability and activity under ORR conditions. Differential charge density analysis provided microscopic insights into electron transfer mechanisms during ORR reactions (Fig. 6h). Upon *OOH intermediate adsorption, both the Mn atom at the adsorption site and the adjacent N_2_O_2_-coordinated Mn atom exhibited electron depletion phenomena, while obvious electron aggregation zones formed between the adsorption site Mn atom and O atom, with significant electron enrichment also occurring around the non-adsorbed oxygen atom of the *OOH intermediate. During *O intermediate adsorption, clear electron aggregation zones appeared above the oxygen atom, while both adsorption site and adjacent site Mn atoms underwent substantial electron transfer. For *OH intermediates, oxygen atoms similarly showed electron enrichment characteristics, with continuous electron transfer from adsorption site and adjacent Mn atoms to intermediates, while slight electron depletion regions appeared around non-metallic coordination atoms surrounding the adsorption site Mn atom. This series of electron transfer processes revealed the synergistic mechanism between adjacent metal centers in dual-atom catalysts, where two Mn atoms jointly participate in ORR reactions through electronic coupling, achieving more efficient electron transfer and reaction activation than single-atom catalysts.

## Conclusion

This work reports the first successful construction of an MM-ON/C diatomic catalyst by replacing conventional symmetric M–N_4_ coordination with an asymmetric N_2_O_4_ design. The catalyst delivers outstanding ORR performance, achieving a half-wave potential of 0.925 V, 65 mV higher than commercial Pt/C—and retaining performance with only an 11 mV loss after 50 000 CV cycles. In zinc–air batteries, it reaches a power density of 209.1 mW cm^−2^ and operates stably for 1000 hours without evident degradation. Crucially, we identify the primary cause of performance decay in MnMn-ON/C after extended cycling as the elevation of Mn oxidation states rather than rupture of coordination bonds. A targeted H_2_/Ar anneal at 500 °C restores 98% of the activity, addressing the long-standing issue of irreversible deactivation in traditional M–N coordinated catalysts. By integrating *in situ* spectroscopy with DFT calculations, we elucidate the cooperative mechanism of Mn dual-atom sites in MnMn-ON/C. Adjacent Mn centers activate O_2_*via* electronic coupling, while mixed N/O coordination lower reaction energy barriers and supports an efficient four-electron transfer pathway. Finally, the oxygen-coordinated multidentate ligand-electrospinning strategy shows strong generality, extending to Co, Cu, and Ni to yield asymmetric diatomic catalysts with controlled atomic spacing. These findings open avenues for exploring diverse metal pairings, coordination environments, and recovery protocols to advance durable, high-activity ORR catalysts.

## Author contributions

Yu Wang conceived and supervised the research, Chuanzhen Feng synthesized the catalysts and conducted performance test, Huijuan Zhang performed DFT calculations. Guangxu Yao analyzed the data and wrote the paper. Yangjun Luo discussed the results and commented on the manuscript.

## Conflicts of interest

The authors declare that they have no known competing financial interests or personal relationships that could have appeared to influence the work reported in this paper.

## Supplementary Material

SC-OLF-D5SC07897K-s001

## Data Availability

The data that support the findings of this study are available from the corresponding authors upon reasonable request. Supplementary information is available. See DOI: https://doi.org/10.1039/d5sc07897k.

## References

[cit1] Wang X., Zhang H., Feng C., Wang Y. (2024). Chem. Sci..

[cit2] Yao G., Luo Y., Wang X., Zheng Y., Guo H., Wang M., Zhang H., Wang Y. (2024). J. Power Sources.

[cit3] Cao Z., Wang C., Sun Y., Liu M., Li W., Zhang J., Fu Y. (2024). Chem. Sci..

[cit4] Yao G., Wan J., Luo Y., Liu D., Qiu G., Zhang H., Wang Y. (2025). J. Catal..

[cit5] Jin Z., Li P., Meng Y., Fang Z., Xiao D., Yu G. (2021). Nat. Catal..

[cit6] Zong L., Lu F., Zhang W., Fan K., Chen X., Johannessen B., Qi D., Bedford N. M., Warren M., Segre C. U., Liu P., Wang L., Zhao H. (2022). Energy Storage Mater..

[cit7] Luo Y., Wang Y., Zhang H., Wang Y., Wan J., Feng C., Liu L., Guo Z., Li J., Wang Y. (2024). Energy Environ. Sci..

[cit8] Cao D., Mu Y., Liu L., Mou Z., Chen S., Yan W., Zhou H., Chan T.-S., Chang L.-Y., Song L., Zhai H.-J., Fan X. (2024). ACS Nano.

[cit9] Bai J., Sun Z., Zhang H., Lian Y., Deng Y., Xiang M., Su Y. (2024). Adv. Funct. Mater..

[cit10] Chen K., Liang Y., Pan D., Huang J., Gao J., Lu Z., Liu X., Chen J., Zhang H., Hu X., Wen Z. (2024). Adv. Energy Mater..

[cit11] Brea C., Hu G. (2023). ACS Catal..

[cit12] Liu S., Li C., Zachman M. J., Zeng Y., Yu H., Li B., Wang M., Braaten J., Liu J., Meyer H. M., Lucero M., Kropf A. J., Alp E. E., Gong Q., Shi Q., Feng Z., Xu H., Wang G., Myers D. J., Xie J., Cullen D. A., Litster S., Wu G. (2022). Nat. Energy.

[cit13] Zhang L., Dong Y., Li L., Shi Y., Zhang Y., Wei L., Dong C.-L., Lin Z., Su J. (2024). Nano-Micro Lett..

[cit14] Li Y., Li Y., Sun H., Gao L., Jin X., Li Y., Lv Z., Xu L., Liu W., Sun X. (2024). Nano-Micro Lett..

[cit15] Yang G., Fan M., Liang Q., He X., Zhang W., Asefa T. (2024). Angew. Chem., Int. Ed..

[cit16] Sun Q., Yue X., Yu L., Li F.-Z., Zheng Y., Liu M.-T., Peng J.-Z., Hu X., Chen H. M., Li L., Gu J. (2024). J. Am. Chem. Soc..

[cit17] Song W.-S., Wang M., Zhan X., Wang Y.-J., Cao D.-X., Song X.-M., Nan Z.-A., Zhang L., Fan F. R. (2023). Chem. Sci..

[cit18] Zhang Z., Xing Z., Luo X., Cheng C., Liu X. (2025). Nat. Commun..

[cit19] Yan Y., Yu R., Liu M., Qu Z., Yang J., He S., Li H., Zeng J. (2025). Nat. Commun..

[cit20] Luo G., Zhu E., Shi C., Ren Y., Lin Y., Yang X., Xu M. (2024). Appl. Catal., B.

[cit21] Sui R., Liu B., Chen C., Tan X., He C., Xin D., Chen B., Xu Z., Li J., Chen W., Zhuang Z., Wang Z., Chen C. (2024). J. Am. Chem. Soc..

[cit22] Zhao Q.-P., Shi W.-X., Zhang J., Tian Z.-Y., Zhang Z.-M., Zhang P., Wang Y., Qiao S.-Z., Lu T.-B. (2024). Nat. Synth..

[cit23] Wu M., Yang X., Cui X., Chen N., Du L., Cherif M., Chiang F.-K., Wen Y., Hassanpour A., Vidal F., Omanovic S., Yang Y., Sun S., Zhang G. (2023). Nano-Micro Lett..

[cit24] Chen Y., Mao J., Zhou H., Xing L., Qiao S., Yuan J., Mei B., Wei Z., Zhao S., Tang Y., Liu C. (2023). Adv. Funct. Mater..

[cit25] Zhang L., Zhang N., Shang H., Sun Z., Wei Z., Wang J., Lei Y., Wang X., Wang D., Zhao Y., Sun Z., Zhang F., Xiang X., Zhang B., Chen W. (2024). Nat. Commun..

[cit26] Shang H., Zhou X., Dong J., Li A., Zhao X., Liu Q., Lin Y., Pei J., Li Z., Jiang Z., Zhou D., Zheng L., Wang Y., Zhou J., Yang Z., Cao R., Sarangi R., Sun T., Yang X., Zheng X., Yan W., Zhuang Z., Li J., Chen W., Wang D., Zhang J., Li Y. (2020). Nat. Commun..

[cit27] Yasin G., Ali S., Ibraheem S., Kumar A., Tabish M., Mushtaq M. A., Ajmal S., Arif M., Khan M. A., Saad A., Qiao L., Zhao W. (2023). ACS Catal..

[cit28] Wang Q., Wang H., Cao H., Tung C.-W., Liu W., Hung S.-F., Wang W., Zhu C., Zhang Z., Cai W., Cheng Y., Tao H. B., Chen H. M., Wang Y.-G., Li Y., Yang H. B., Huang Y., Li J., Liu B. (2023). Nat. Catal..

[cit29] Ji S., Mou Y., Liu H., Lu X., Zhang Y., Guo C., Sun K., Liu D., Horton J. H., Wang C., Wang Y., Li Z. (2024). Adv. Mater..

[cit30] Liu H., Tian L., Zhang Z., Wang L., Li J., Liang X., Zhuang J., Yin H., Yang D., Zhao G., Su F., Wang D., Li Y. (2024). J. Am. Chem. Soc..

[cit31] Yin L., Sun M., Zhang S., Huang Y., Huang B., Du Y. (2024). Adv. Mater..

[cit32] Rao P., Han X., Sun H., Wang F., Liang Y., Li J., Wu D., Shi X., Kang Z., Miao Z., Deng P., Tian X. (2024). Angew. Chem., Int. Ed..

[cit33] Chao G., Zhang Y., Zhang L., Zong W., Zhang N., Xue T., Fan W., Liu T., Xie Y. (2022). J. Mater. Chem. A.

[cit34] Zhang J., Li F., Liu W., Wang Q., Li X., Hung S. F., Yang H., Liu B. (2024). Angew. Chem., Int. Ed..

[cit35] Ji S. G., Kim M. M., Han M. H., Cho J., Son Y., Kim Y. Y., Jeong J., Kim Z. H., Chae K. H., Oh H.-S., Kim H., Choi C. H. (2024). Nat. Catal..

[cit36] Gao R., Wang J., Huang Z.-F., Zhang R., Wang W., Pan L., Zhang J., Zhu W., Zhang X., Shi C., Lim J., Zou J.-J. (2021). Nat. Energy.

[cit37] Huang Z., Li M., Yang X., Zhang T., Wang X., Song W., Zhang J., Wang H., Chen Y., Ding J., Hu W. (2024). J. Am. Chem. Soc..

[cit38] Wang Z., Jin X., Zhu C., Liu Y., Tan H., Ku R., Zhang Y., Zhou L., Liu Z., Hwang S. J., Fan H. J. (2021). Adv. Mater..

[cit39] Zhang X., Liu X., Wu D., Hu L., Zhang H., Sun Z., Qian S., Xia Z., Luo Q., Cao L., Yang J., Yao T. (2024). Nano Lett..

[cit40] Qu Q., Mao Y., Ji S., Liao J., Dong J., Wang L., Wang Q., Liang X., Zhang Z., Yang J., Li H., Zhou Y., Wang Z., Waterhouse G. I. N., Wang D., Li Y. (2025). J. Am. Chem. Soc..

[cit41] Liu M., Zhang J., Su H., Jiang Y., Zhou W., Yang C., Bo S., Pan J., Liu Q. (2024). Nat. Commun..

[cit42] Zhao Y., Gao Z., Zhang S., Guan X., Xu W., Liang Y., Jiang H., Li Z., Wu S., Cui Z., Zhu S. (2025). Adv. Funct. Mater..

