# Supplementary material for: A universal N2O4-cavity strategy for precisely spaced, durable dual-atom ORR catalysts
Source: Chem Sci. 2025 Oct 31;16(48):23064–76. doi: 10.1039/d5sc07897k (PMC12577015; doi:10.1039/d5sc07897k)
Supplement: SC-016-D5SC07897K-s001 [file SC-016-D5SC07897K-s001.pdf]

Supplementary Information (SI) for Chemical Science.
This journal is © The Royal Society of Chemistry 2025

A Universal N_2O_4-Cavity Strategy for Precisely Spaced, Durable Dual-Atom ORR Catalysts

Guangxu Yao [a], Huijuan Zhang [a*], Yangjun Luo [b], Chuanzhen Feng [a], and Yu Wang [a*]

[a] *State Key Laboratory of Power Transmission Equipment Technology, School of Chemistry and Chemical Engineering, Chongqing University, Chongqing, 400044, P. R. China.*

[b] *BYD Automobile Industry Co. Ltd., Shenzhen, Guangdong Province 518118, PR China*

[*] *E-mail: wangy@cqu.edu.cn; zhanghj@cqu.edu.cn*

Experimental Section

Materials

All reagents used in this study were of analytical grade and required no further purification. 2-hydroxy-3-methoxybenzaldehyde ($C_8H_8O_3$, 99%) and ethylenediamine ($C_2H_8N_2$, 98%) were purchased from Aladdin Reagent Co., Ltd. Copper nitrate ($Cu(NO_3)_2$, 99%), manganese nitrate ($Mn(NO_3)_2$, 98%), nickel nitrate ($Ni(NO_3)_2$, 98%), cobalt nitrate ($Co(NO_3)_2$, 98%), anhydrous ethanol, anhydrous methanol, polyvinylpyrrolidone (PVP), phthalocyanine, DMF, g-C_3N_4, polyacrylonitrile (PAN), dichloromethane, and n-hexane were obtained from Shanghai Macklin Biochemical Technology Co., Ltd.

Catalyst Preparation

Synthesis of Catalyst Precursor H_2L

Ethylenediamine and 2-hydroxy-3-methoxybenzaldehyde were mixed in a 1:2 molar ratio (mmol) and refluxed in anhydrous ethanol at 50–55°C for 5 hours. Upon completion, a clear solution was obtained. The solvent was removed under reduced pressure using a rotary evaporator at 50°C. The resulting product was recrystallized in a dichloromethane and n-hexane mixture (1:10, v/v) to yield a yellow solid, 6,6'-((1E,1'E)-(ethane-1,2-diylbis(azaneylylidene))bis(methaneylylidene))bis(2-methoxyphenol) (H_2L), with a total yield of 69%. The product, identified as $C_{18}H_{20}N_2O_4$, was characterized through elemental analysis, ^{1}H NMR spectroscopy, and mass spectrometry. Theoretical elemental analysis: C, 65.84%; H, 6.14%; N, 8.53%; O, 19.49%. Found: C, 65.80%; H, 6.18%; N, 8.50%; O, 19.52%. 1H NMR (500 MHz, Chloroform-d) revealed peaks at δ 13.59 (s, 2H), 8.32 (s, 2H), 6.89 (dd, J = 7.9, 1.6 Hz, 2H), 6.84 (dd, J = 7.9, 1.6 Hz, 2H), 6.77 (t, J = 7.8 Hz, 2H), 3.95 (s, 4H), and 3.88 (s, 6H). High-resolution mass spectrometry (HRMS) confirmed the molecular

composition of $C_{18}H_{20}N_2O_4$, with a calculated $[H_2L-H^+]^+$ of 327.14 and a measured value of 327.13, as shown in Fig. S2a and Fig. S2b.

Synthesis of Catalysts MnMn-ON/C, MnMn-N/C, Mn-N/C, ON/C, N/C, NiNi-ON/C, CoCo-ON/C, and CuCu-ON/C

Using MnMn-ON/C as an example, the synthesis proceeds in three main stages. First, 0.01 mmol of H_2L is dissolved in dichloromethane and stirred for 5 min. Separately, 0.02 mmol of manganese nitrate is dissolved in ethanol and stirred for 5 min. The two solutions are then combined, an obvious color change occurs, and stirring is continued for 10 min to promote coordination between the ligand and the metal ions. The mixture is filtered to obtain a clear solution. In the second stage, 500 mg polyvinylpyrrolidone (PVP), 500 mg polyacrylonitrile (PAN), and appropriate amounts of ethanol and DMF are added to this solution to yield a pale, viscous mixture suitable for electrospinning. Third, the solution is loaded into a metallic syringe fitted with a single nozzle, the flow rate is set to 1.5 mL h^{-1}, and electrospinning is initiated. A polished aluminum plate is placed 20 cm from the needle tip, and a 19 kV DC high voltage is applied between the nozzle and the plate. After electrospinning, the resulting white membrane is carefully removed, heated at 75 °C for 1.5 h to evaporate residual solvent, then heated in air to 180 °C at 10 °C min^{-1} and held for 1 h, and finally heated in an argon atmosphere to 600 °C at 5 °C min^{-1} and held for 1.5 h. NiNi-ON/C, CoCo-ON/C, and CuCu-ON/C are prepared by the same procedure, simply replacing the corresponding metal nitrate. MnMn-N/C is synthesized via the widely used g-C_3N_4 route[1-2]. In the first step, 5 mg g-C_3N_4 is ultrasonically dispersed in 5 mL DMF, after which 0.02 mmol manganese nitrate and 0.04 mmol 2-methylimidazole are added, and the mixture is sonicated for 30 min. The second and third steps are identical to those for MnMn-ON/C. Mn-N/C is obtained by combining manganese ions with phthalocyanine followed by annealing. MnMn-ON/C-Air is produced by annealing MnMn-ON/C in air. For N/C, phthalocyanine is annealed directly.

Naming Rules for -Air, -CV, -HA, -A, etc.

For ease of discussion, samples processed under different conditions are named according to the following rules: Samples after cyclic voltammetry (CV) are marked as "CV + number of cycles" (e.g., CV1W represents 10,000 cycles), samples treated with H2/Ar annealing are marked as "HA + temperature" (e.g., HA500 represents hydrogen annealing at 500°C), air-annealed samples are marked as "Air", and Ar atmosphere-annealed samples are marked as "A + temperature".

The Pt/C catalyst after 10,000 and 50,000 CV cycles is named Pt/C-CV1W and Pt/C-CV5W, respectively. The MnMn-N/C catalyst after 10,000 and 50,000 CV cycles is named MnMn-N/C-CV1W and MnMn-N/C-CV5W, respectively. The MnMn-ON/C catalyst after 50,000 CV cycles is named MnMn-ON/C-CV5W. MnMn-ON/C-Air refers to the sample prepared by annealing in air instead of Ar atmosphere. After holding the MnMn-ON/C-CV5W in H2/Ar atmosphere at 300°C, 400°C, and 500°C for 40 minutes, they are named MnMn-ON/C-CV5W-HA300, MnMn-ON/C-CV5W-HA400, and MnMn-ON/C-CV5W-HA500, respectively. After holding the MnMn-ON/C-Air in H2/Ar atmosphere at 300°C, 400°C, and 500°C for 40 minutes, they are named MnMn-ON/C-Air-HA300, MnMn-ON/C-Air-HA400, and MnMn-ON/C-Air-HA500, respectively. After holding the MnMn-ON/C-CV5W in Ar atmosphere at 300°C, 400°C, and 500°C for 40 minutes, they are named MnMn-ON/C-CV5W-A300, MnMn-ON/C-CV5W-A400, and MnMn-ON/C-CV5W-A500, respectively.

Catalyst Characterization

X-ray photoelectron spectroscopy (XPS, ESCALAB 250Xi, Thermo Scientific) was used to analyze the chemical states and elemental composition on the catalyst surface by measuring the energy of photoelectrons emitted from the sample surface. Transmission electron microscopy (TEM FEI Titan ChemiSTEM) was utilized to observe the microstructure of the materials, providing atomic-level images for analyzing the crystal structure and defects of the materials. Inductively coupled plasma optical emission spectrometry (ICP-OES, ICP-OES 6300, Thermo Fisher Scientific)

was employed for the precise determination of metal and non-metal elemental compositions in the materials by exciting the elements within the sample and measuring the intensity of emitted light. Field emission scanning electron microscopy (SEM, JEOL JSM-7800F) was used to observe the external morphology and microstructure of the materials, offering high-resolution images of the sample surface. Nitrogen adsorption-desorption isotherm analysis (Microtrac BEL, BET system) was conducted to evaluate key parameters such as pore size, pore volume, and specific surface area by measuring the adsorption and desorption of nitrogen on the material surface. X-ray diffraction (XRD, PANalytical X'Pert Powder) was applied to determine the crystal structure of the materials by analyzing their diffraction patterns to identify phase composition and crystal orientation. Energy dispersive X-ray spectroscopy (EDS), coupled with electron microscopy, was used to identify the elements present in the sample and their contents, relying on characteristic X-rays emitted under electron beam irradiation. Synchrotron radiation data were obtained at the XAFS station of beamline 14W1 at the Shanghai Synchrotron Radiation Facility. For the organic compound H_2L, ^1H NMR spectra were recorded using a Bruker AVANCE DRX-400 nuclear magnetic resonance spectrometer. Electrospray ionization mass spectra were acquired with a Bruker Daltonics Esquire 6000 electrospray ionization mass spectrometer. Raman spectroscopic data were employed an XploRA PLUS instrument with a 532 nm wavelength excitation source. In situ Raman spectroscopic measurements, a WITec alpha 300R confocal system was utilized, which incorporated a 633 nm excitation beam, coupled with a Zeiss 50× magnification objective and a diffraction element containing 600 grooves/mm. In situ ATR-SEIRAS measurements were conducted at the Shanghai Synchrotron Radiation Facility, using a Fourier Transform Infrared Spectrometer for analysis. The experimental setup included a potassium bromide beam splitter, an MCT detector, and a Bruker Hyperion 3000 external microscope, employing a reflection mode with vertically incident infrared light. The spectral detection range covered 700–3600 cm^{-1}, with a resolution of 0.25 cm^{-1}. To effectively suppress interference caused by water molecule vibrational absorption bands, the research team selected a zinc selenide crystal window and tightly coupled the catalyst electrode to the

window, minimizing infrared light transmission losses. The experiment utilized a standard three-electrode system with 0.1 M potassium hydroxide saturated with oxygen as the electrolyte, circulated via a peristaltic pump. The testing procedure involved initially acquiring the background spectrum of the catalyst electrode, followed by systematic measurements of the oxygen reduction reaction (ORR). The potential range was set from 0.65 to 1 V, with each measurement preceded by a 20-minute stabilization at the specific potential to ensure electrode surface stability.

Electrochemical Measurements

Catalyst ink preparation and electrochemical testing conditions: Catalyst ink was prepared by mixing 1.0 mg of catalyst with 20 μL isopropanol, 20 μL of 5 wt% Nafion solution and 160 μL ultrapure water, followed by sonication for 60 min to obtain a homogeneous suspension. 15 μL of the ink was carefully drop-cast onto a polished and cleaned rotating ring-disk electrode (RRDE, disk diameter 5.0 mm), resulting in a catalyst loading of 0.38 mg cm^{-2}. The electrode was left to dry at room temperature for 60 min prior to measurements. Electrochemical experiments were performed in a three-electrode configuration (AFMSRCE, PINE, USA) with the catalyst-loaded RRDE as the working electrode, a reversible hydrogen electrode (RHE) as the reference electrode, and a graphite rod as the counter electrode (1). All potentials are reported versus RHE and all data were IR-compensated. ORR measurements were conducted in O_2-saturated 0.1 M KOH. Linear sweep voltammetry (LSV) was recorded over the potential range 0.20–1.10 V vs. RHE at a scan rate of 5 mV s^{-1}. To stabilize the electrode surface, the electrode was pre-cycled 50 times at 50 mV s^{-1} before data acquisition. LSVs were collected at rotation rates of 400, 800, 1200, 1600, 2000 and 2400 rpm for Koutecky–Levich analysis and determination of the electron transfer number (2). In RRDE measurements the ring potential was held at 1.50 V vs. RHE to detect peroxide species; the collection efficiency of the Pt ring (N) is 0.38. The calculation methods for the electron transfer number (n) and H_2O_2 yield are given in Equations (3)–(6) of the main text. Stability tests included chronoamperometry (CA) at

0.70 V vs. RHE to evaluate long-term current retention, and cyclic voltammetry (CV) cycling between 0.60–1.00 V vs. RHE at 100 mV s^{-1}. Methanol tolerance tests were performed by adding 3.0 M methanol to 0.1 M KOH and monitoring the current at 0.70 V vs. RHE by chronoamperometry. All electrochemical measurements were carried out at room temperature (~25 °C); prior to measurements the electrolyte was purged with high-purity O_2 (or N_2 for background scans) for at least 30 min to ensure saturation.

The catalyst ink was prepared by thoroughly mixing 1 mg of catalyst with 20 μL of isopropanol, 20 μL of 5 wt% Nafion solution, and 160 μL of ultrapure water, followed by ultrasonication for 60 minutes. Subsequently, 15 μL of the prepared ink was carefully drop-cast onto a pre-polished and cleaned rotating ring-disk electrode (RRDE), achieving a catalyst loading of 0.38 mg cm^{-2}. The electrode was then air-dried at room temperature for 60 minutes before testing. Electrochemical experiments were conducted using a three-electrode system (AFMSRCE, PINE, USA). A mercury/mercury oxide electrode served as the reference electrode, a graphite electrode as the counter electrode, and the catalyst-loaded RRDE as the working electrode. All potentials reported in this study were calibrated to the reversible hydrogen electrode (RHE) using Equation (1), and all data were corrected for IR compensation. Oxygen reduction reaction (ORR) tests were performed in a 0.1 M potassium hydroxide solution. To obtain stable linear sweep voltammetry (LSV) curves, the electrode was cycled 50 times at a scan rate of 50 mV s^{-1}. Some experimental parameters were calculated using the Koutecky-Levich (K-L) equation. In Equation (2), J represents the current density, JL the limiting diffusion current density, JK the kinetic current density, B the reciprocal of the K-L slope, and ω the angular velocity. In Equation (3), n, F, C_0,

v, and D_0 denote the number of electrons transferred, Faraday constant (96485.33 C mol^{-1}), oxygen bulk concentration (1.2×10^{-6} mol cm^{-3}), kinematic viscosity of the electrolyte (0.01 cm^2 s^{-1}), and oxygen diffusion coefficient (1.9×10^{-5} cm^2 s^{-1}), respectively. The H_2O_2 yield during ORR was calculated using Equation (4), while the electron transfer number was determined via Equation (5), where Ir is the ring current, Id the disk current, and N the current collection efficiency of the platinum ring (0.38). The Tafel slope was derived from Equation (6), where η is the overpotential, a the overpotential at unit current density, b the Tafel slope, j the current density, and j_0 the exchange current density.

$$E^{(RHE)} = E^{\theta} + 0.0591pH + 0.098 \quad (1)$$

$$\frac{1}{J} = \frac{1}{J_L} + \frac{1}{J_K} = \frac{1}{B\omega^{\frac{1}{2}}} + \frac{1}{J_K} \quad (2)$$

$$B = 0.2nFC_0 v^{\frac{1}{6}} (D_0)^{\frac{2}{3}} \quad (3)$$

$$H_2O_2(\%) = \frac{200Ir/N}{Ir/N + I_d} \quad (4)$$

$$n = \frac{4I_d}{Ir/N + I_d} \quad (5)$$

$$\eta = a + b\log(\frac{j}{j_0}) \quad (6)$$

Zinc-Air Battery Evaluation

Conventional rechargeable liquid zinc-air batteries were assembled as follows. A mixture of 2 mg catalyst, 40 μL of 5 wt% Nafion solution, and 360 μL of anhydrous ethanol was ultrasonically dispersed. The resulting suspension was uniformly coated onto PTFE-modified carbon paper, serving as the cathode material. A polished zinc

metal plate (0.25 mm thick) was used as the anode. The electrolyte was prepared by mixing 6 mol/L KOH solution with 0.2 mol/L zinc acetate solution. Nickel foam, matching the zinc plate's dimensions, was employed as the current collector. The components were assembled in sequence to form a complete zinc-air battery. For comparison, a 1:1 mass ratio mixture of Pt/C and RuO_2 was used as a reference catalyst. Long-term cycling tests were conducted at a constant charge-discharge current density of 5 mA cm^{-1}, with each cycle lasting 20 minutes. The specific capacity of the battery was calculated by recording the mass of zinc foil consumed during continuous discharge at 5 mA cm^{-2}, using the formula: specific capacity = current × test time / mass of consumed zinc.

Flexible solid-state zinc-air batteries were prepared as follows. A polished zinc foil (0.1 mm thick) served as the anode, while catalyst-loaded carbon cloth combined with nickel foam formed the air electrode. The gel electrolyte was prepared by dispersing 7 g of polyvinyl alcohol (PVA) powder in 40 mL of ultrapure water, followed by continuous stirring at 93°C for 2 hours to ensure complete dissolution of PVA. Subsequently, 20 mL of the electrolyte used in the liquid zinc-air battery was added, and stirring continued for 30 minutes to achieve uniformity. After cooling, the mixture was refrigerated for 2 hours to form a gel electrolyte. The flexible solid-state zinc-air battery was assembled by placing a gel electrolyte sheet, matching the zinc foil's dimensions, between the zinc foil and the air electrode, followed by securing the components. Long-cycle testing was performed at a constant charge-discharge current density of 2 mA cm^{-1}, with each cycle lasting 20 minutes.

Computational Methods

Density functional theory (DFT) computations were carried out using the Vienna Ab initio Simulation Package (VASP) [3-7]. Ion-electron interactions were modeled using the projector augmented wave (PAW) method and pseudopotentials. Electron-electron interactions were addressed within the framework of the Perdew-Burke-Ernzerhof (PBE) generalized gradient approximation (GGA). A vacuum layer of 20 Å

was introduced above the slab surface, followed by structural optimization where lattice parameters for the slab configuration were relaxed. All calculations incorporated spin polarization, utilizing a 2×2×1 k-point mesh with a plane-wave cutoff energy of 500 eV. The energy convergence criterion was established at 1×10^{-5} eV, and the force convergence threshold was set to 0.04 eV $Å^{-1}$. Structural visualizations were generated with VESTA, while post-processing was performed using the vaspkit toolkit.

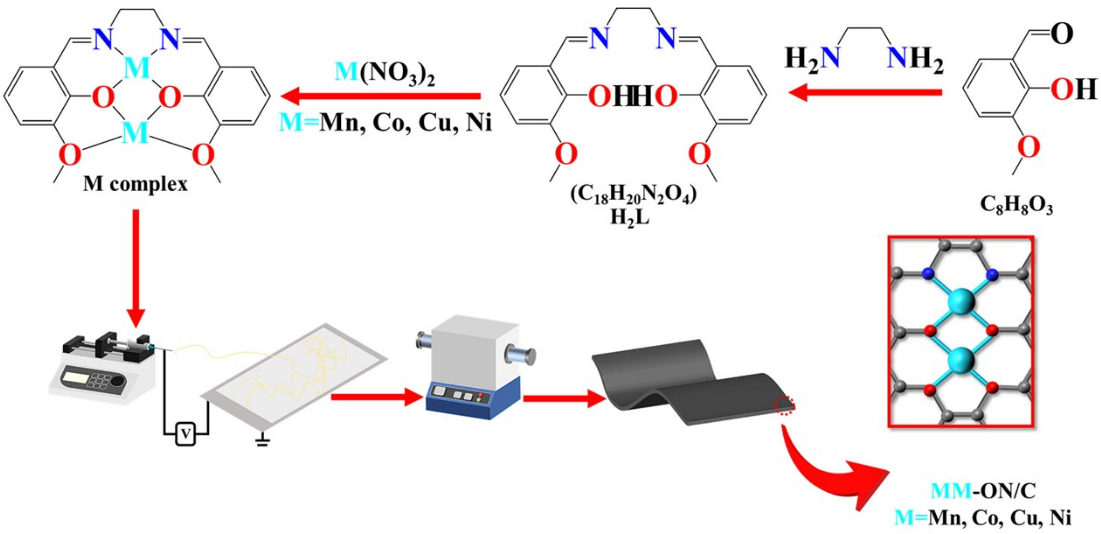

Fig. S1 Detailed synthetic route of the MM-ON/C series catalysts.

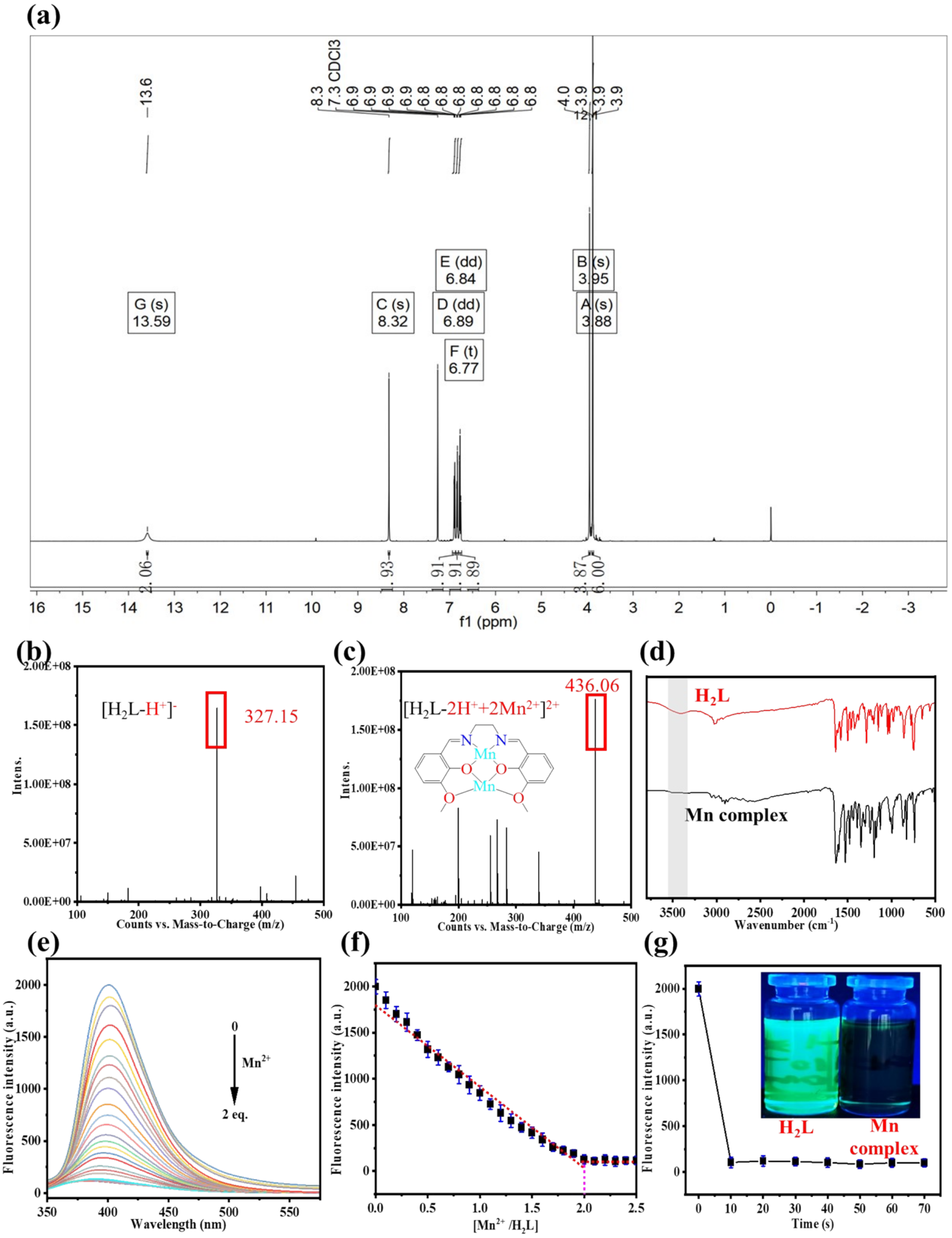

Fig. S2 (a) ^{1}HNMR spectrum of H_2L. (b) Mass Spectrometry of H_2L. (c) Mass Spectrometry of Mn complex. (d) IR spectra of H_2L and Mn complex. (e) Fluorescence spectrum of H_2L (fixed at 1 equivalent), showing the change in solution fluorescence intensity upon the addition of Mn^{2+} (molar equivalents from 0 to 2 eq.). (f) Quantitative Titration Curve of Fluorescence Intensity vs. Mn^{2+}/H_2L Molar Ratio. (g) Stability curve of fluorescence intensity over time; the inset provides a visual comparison of the fluorescence intensity of H_2L and the Mn complex under UV light.

(a) (b)

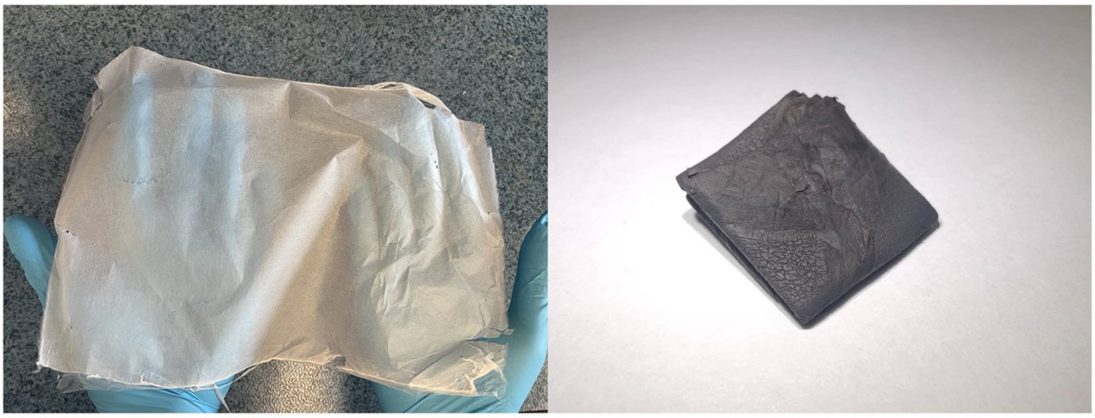

Fig. S3 (a). State of the electrospun material after drying, before annealing (Optical photograph). (b). State of the electrospun material after drying, after annealing, demonstrating its flexibility by folding (Optical photograph).

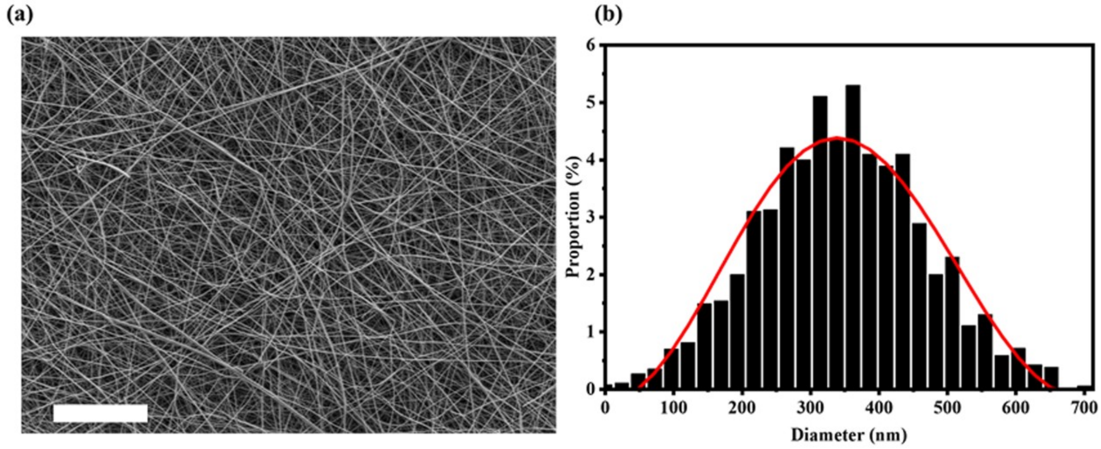

Fig. S4 (a). SEM image of MnMn-ON/C (scale bar: 10 μm). (b). Corresponding fiber diameter distribution.

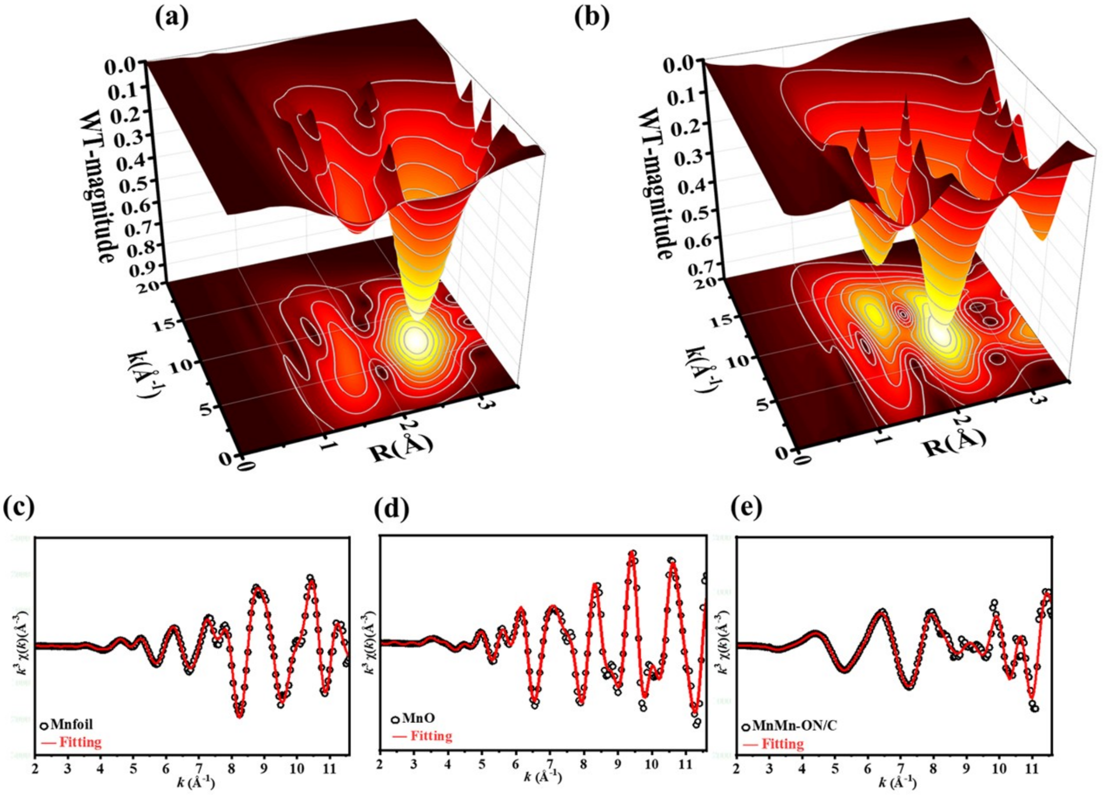

Fig. S5. (a) Wavelet transform image of Mn in MnO. (b) Wavelet transform image of Mn in Mn foil. (c) EXAFS fitting curve of Mn foil in k-space. (d) EXAFS fitting curve of MnO in k-space. (e) EXAFS fitting curve of MnMn-ON/C in k-space.

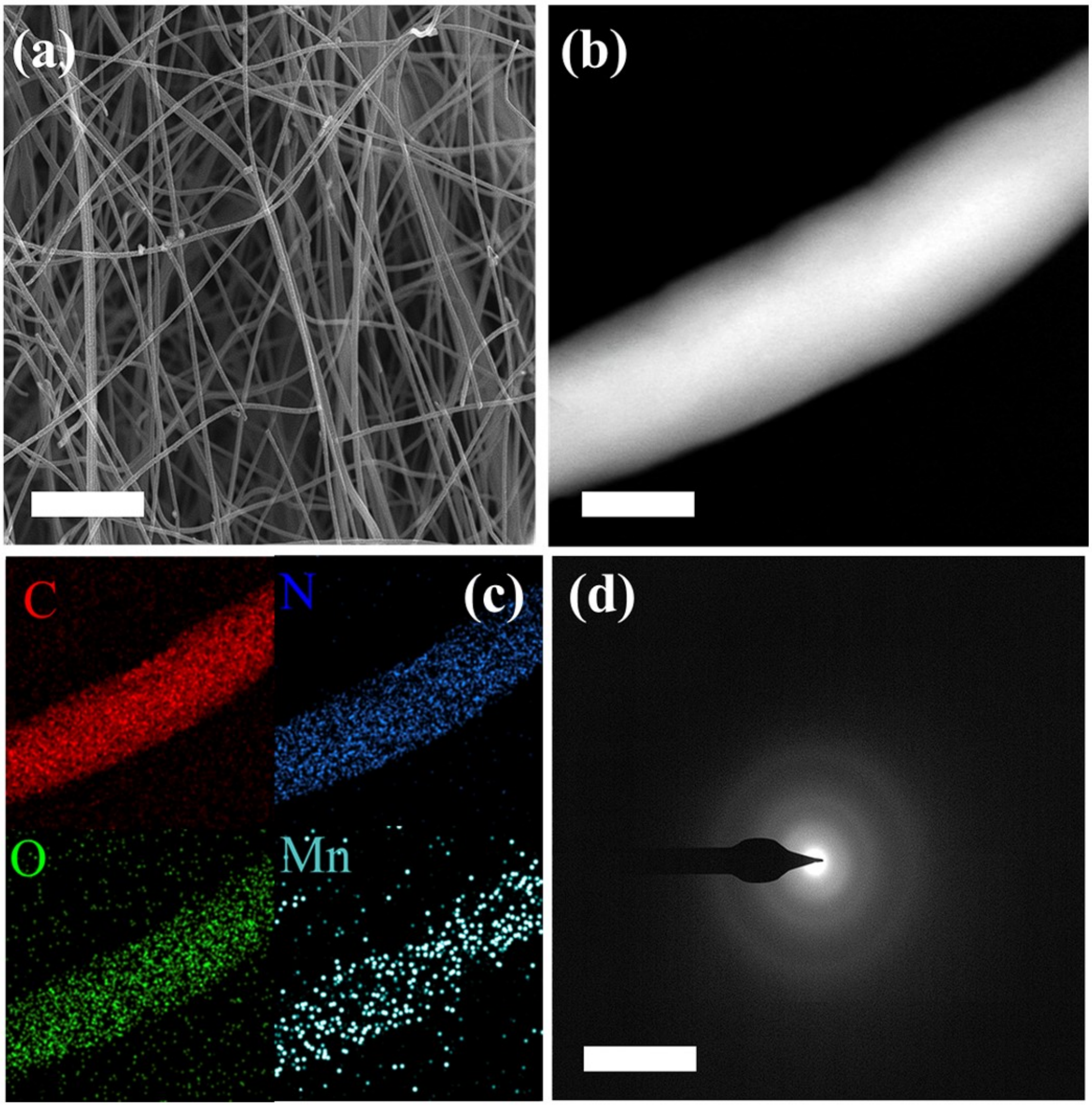

Fig. S6 (a) SEM image of MnMn-N/C (2 μm). (b) HAADF STEM image of MnMn-N/C (500 μm). (c) Elemental distribution map of MnMn-N/C. (d) SAED image of catalyst MnMn-N/C (5 1/nm).

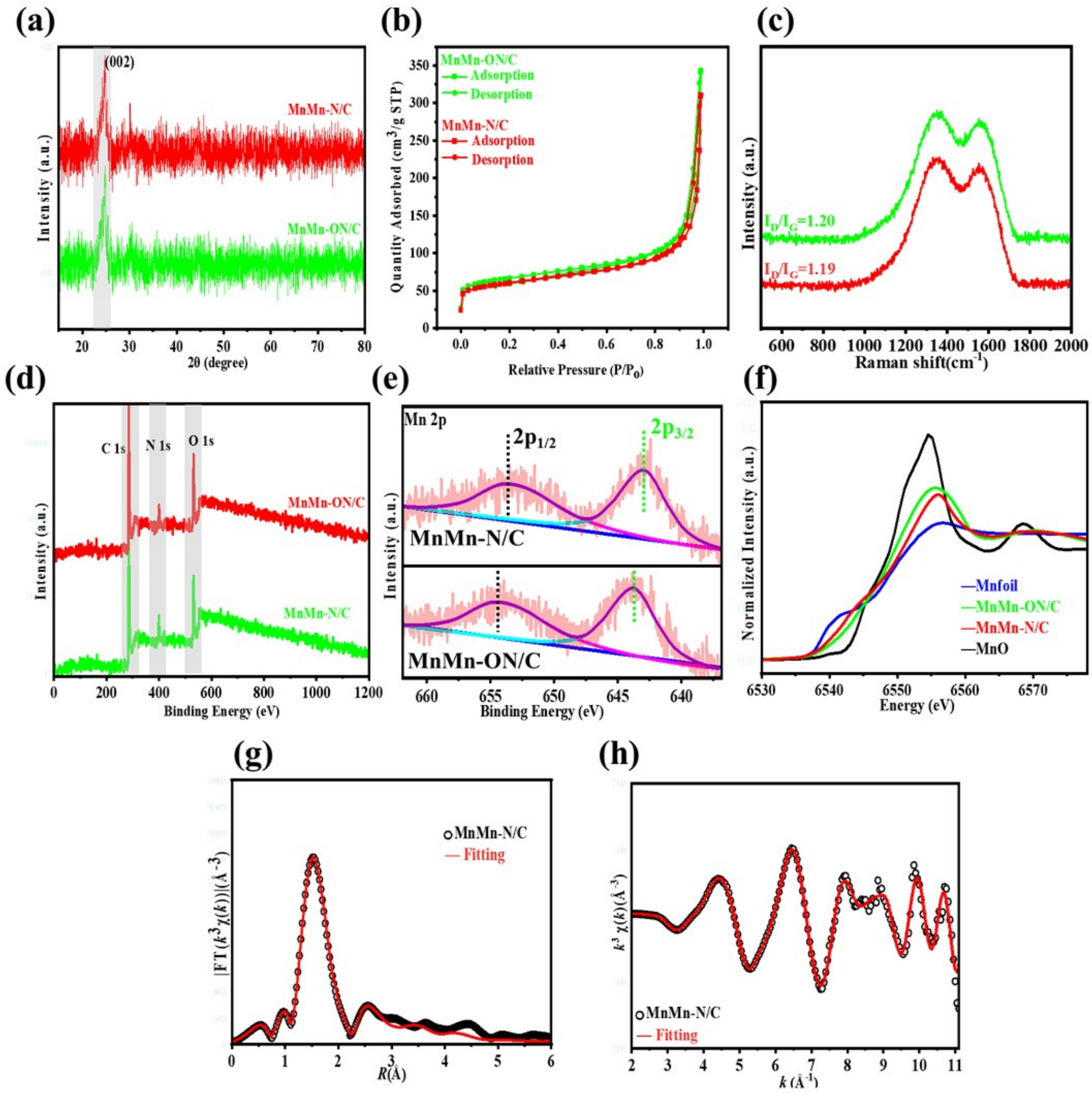

Fig. S7 (a) XRD patterns of catalysts MnMn-N/C and MnMn-ON/C. (b) BET patterns of catalysts MnMn-N/C and MnMn-ON/C. (c) Raman spectra of MnMn-N/C (red) and MnMn-ON/C (cyan). (d) Survey XPS spectra of catalysts MnMn-N/C and MnMn-ON/C. (e) Mn 2p spectra of catalysts MnMn-N/C and MnMn-ON/C. (f) Mn K-edge XANES of Mn foil, MnO, MnMn-N/C, and MnMn-ON/C. (g) Fourier-transformed EXAFS spectrum of MnMn-N/C. The red solid line represents the Mn fitted spectrum of MnMn-N/C. (h) EXAFS fitting curve of MnMn-N/C in k-space.

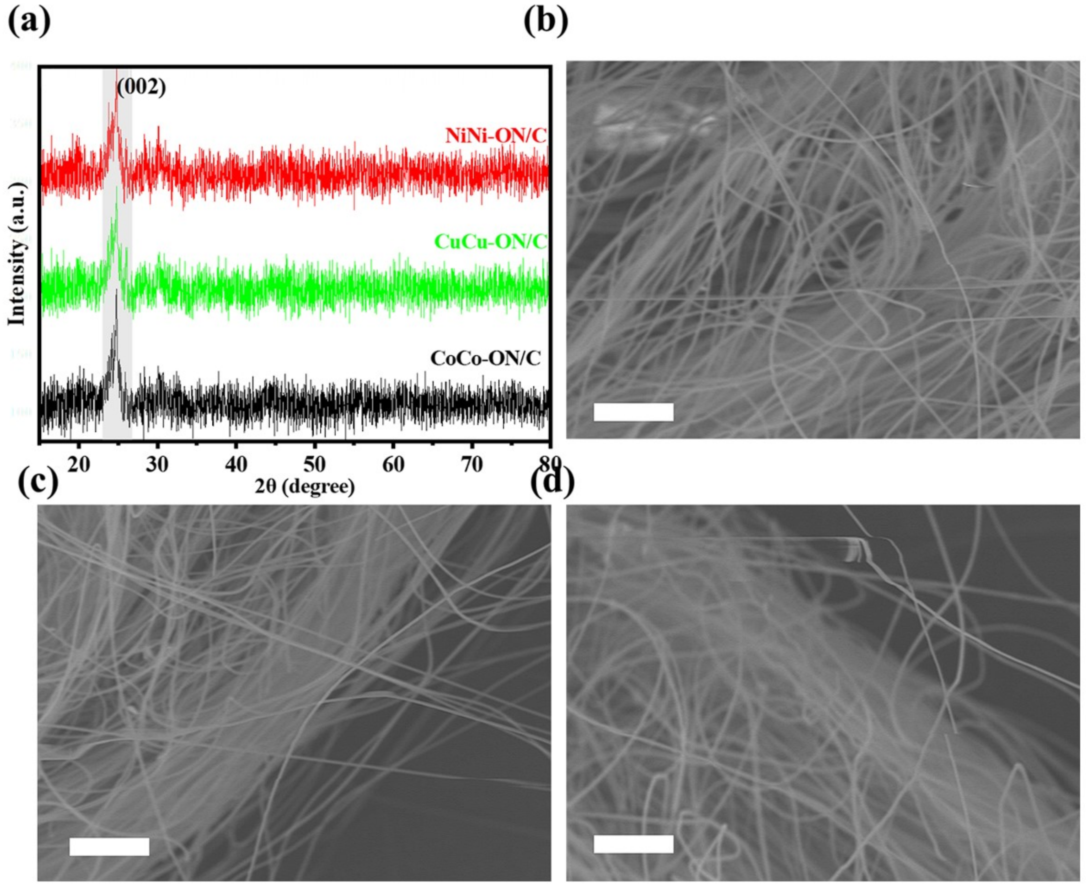

Fig. S8 (a) XRD images of catalysts NiNi-ON/C, CuCu-ON/C, and CoCo-ON/C. (b) SEM image of catalyst NiNi-ON/C (10μm). (c) SEM image of catalyst CuCu-ON/C-ON/C (10μm). (d) SEM image of catalyst CoCo-ON/C (10μm).

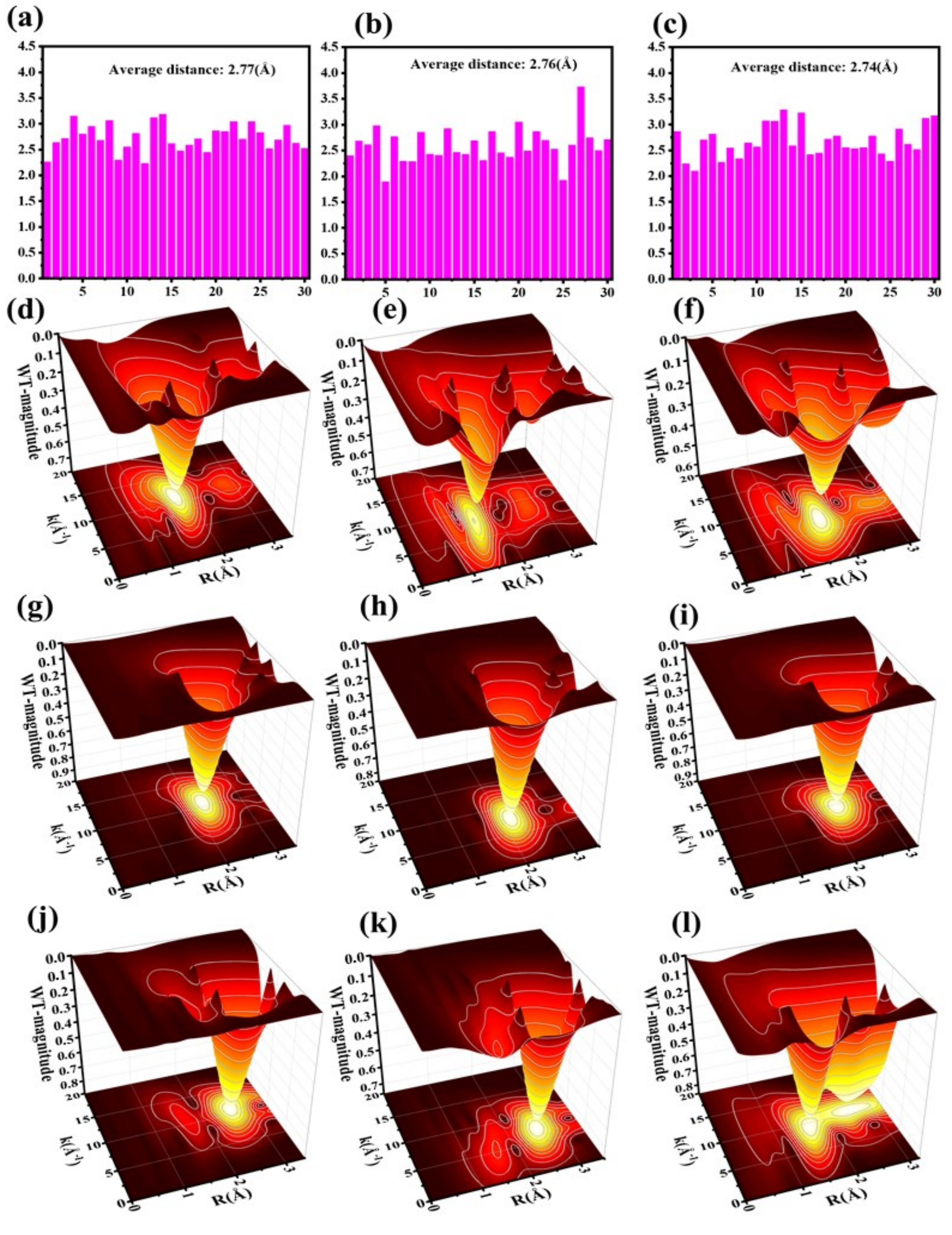

Fig. S9 (a), (b), and (c) show the statistical distances between Ni-Ni, Co-Co, and Cu-Cu diatomic atomic sites. (d) to (l) are wavelet transform images: (d) NiNi–ON/C. (e) CoCo–ON/C. (f) CuCu–ON/C. (g) Ni foil. (h) Co foil. (i) Cu foil. (j) NiO. (k) CoO. (l) CuO.

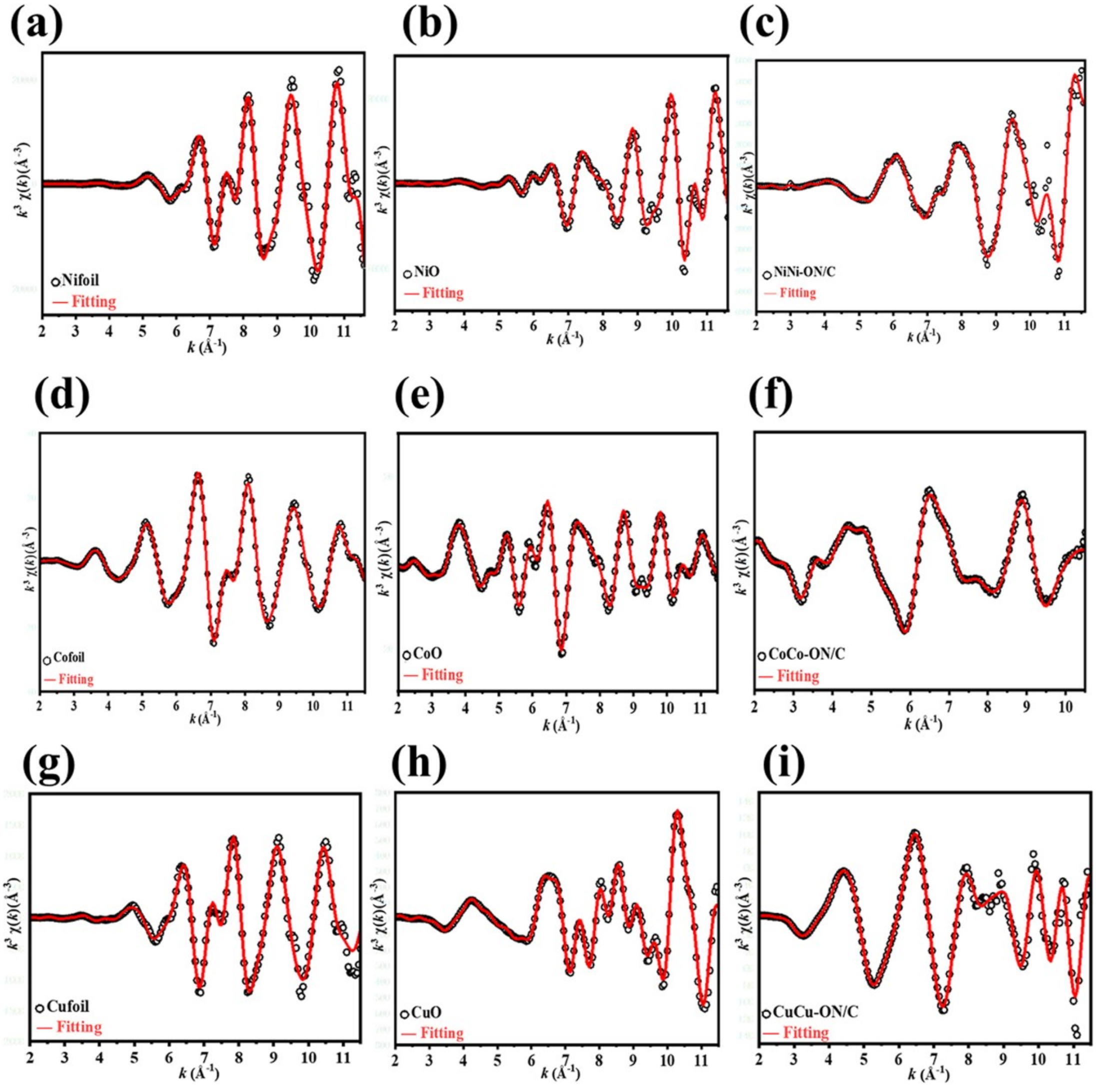

Fig. S10 EXAFS fitting curves in k-space for metal elements in several samples. (a) Ni foil. (b) NiO. (c) NiNi-ON/C. (d) Co foil. (e) CoO. (f) CoCo-ON/C. (g) Cu foil. (h) CuO. (i) CuCu-ON/C.

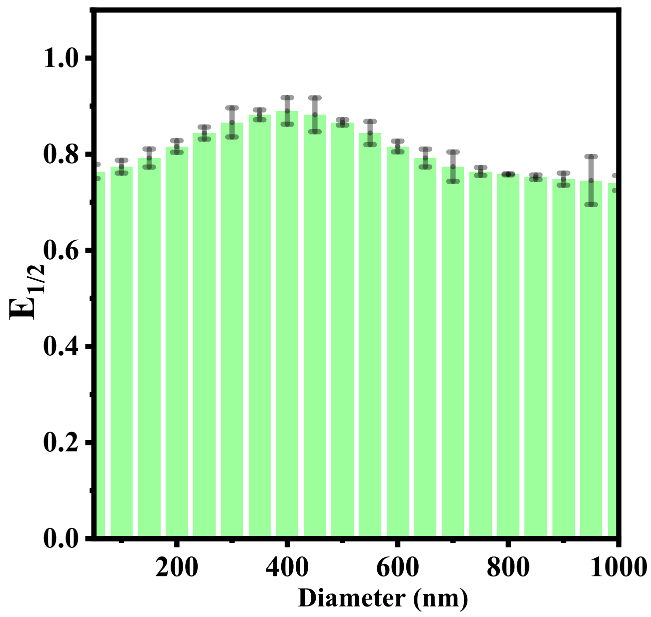

Fig. S11. Relationship between half-wave potential and fiber diameter, multiple measurements with error shown.

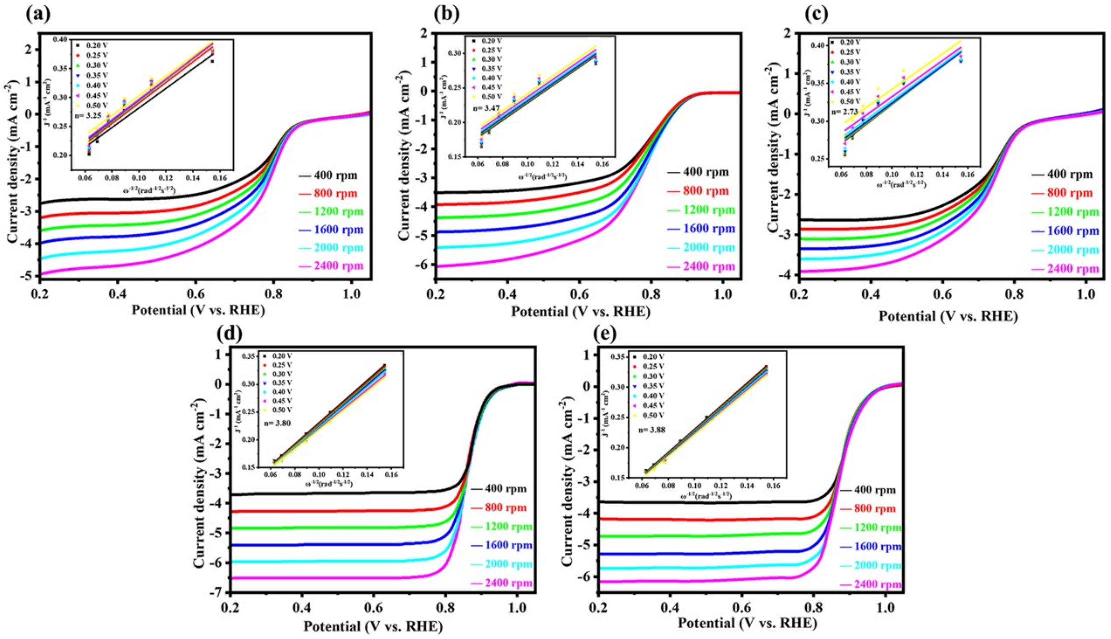

Fig. S12. Relationship between current density and rotation speed for several catalysts, with the inset showing the calculated electron transfer number. (a) MnMn-ON/C-Air. (b) Mn-N/C. (c) N/C. (d) MnMn-N/C. (e) Pt/C.

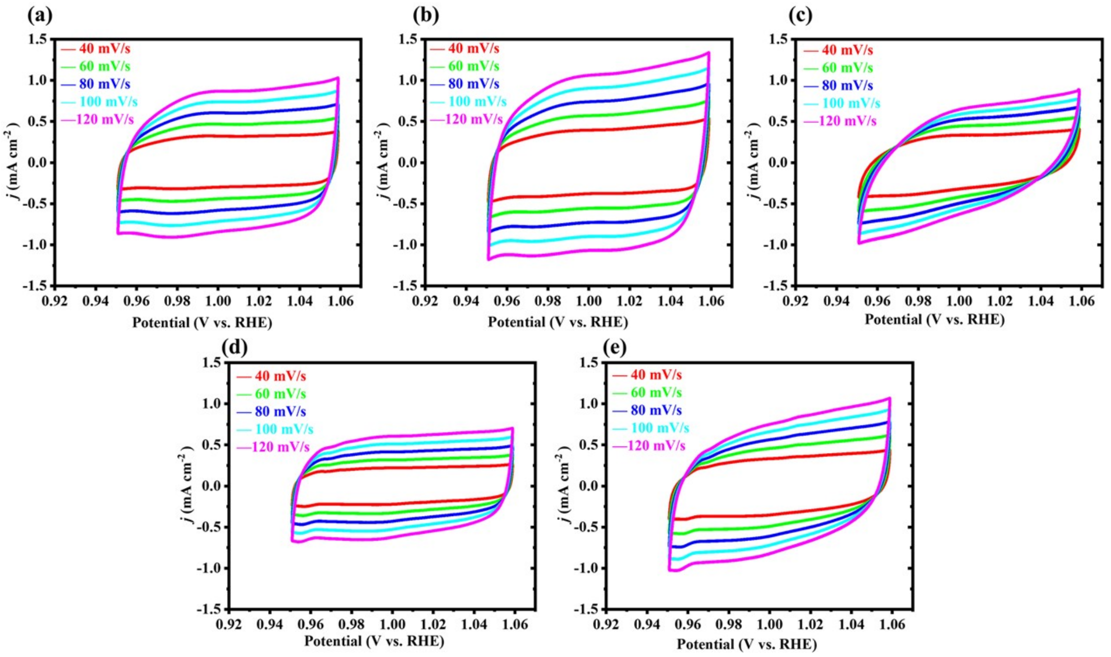

Fig. S13 CV curves of several samples. (a) MnMn-N/C. (b) MnMn-ON/C. (c) N/C. (d) MnMn-ON/C-Air. (e) Mn/NC.

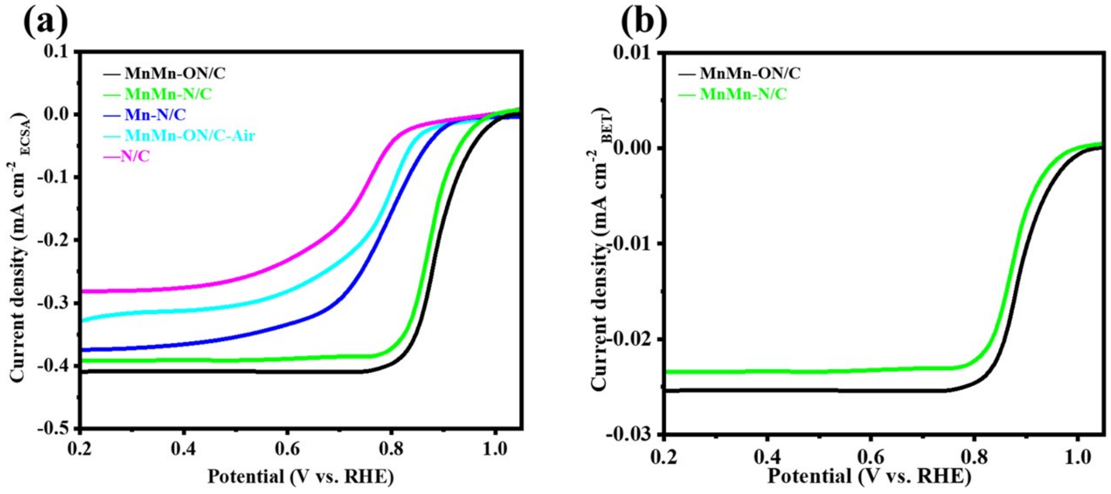

Fig. S14 (a) LSV curves normalized by ECSA comparing catalyst performance. (b) LSV curves normalized by BET comparing catalyst performance.

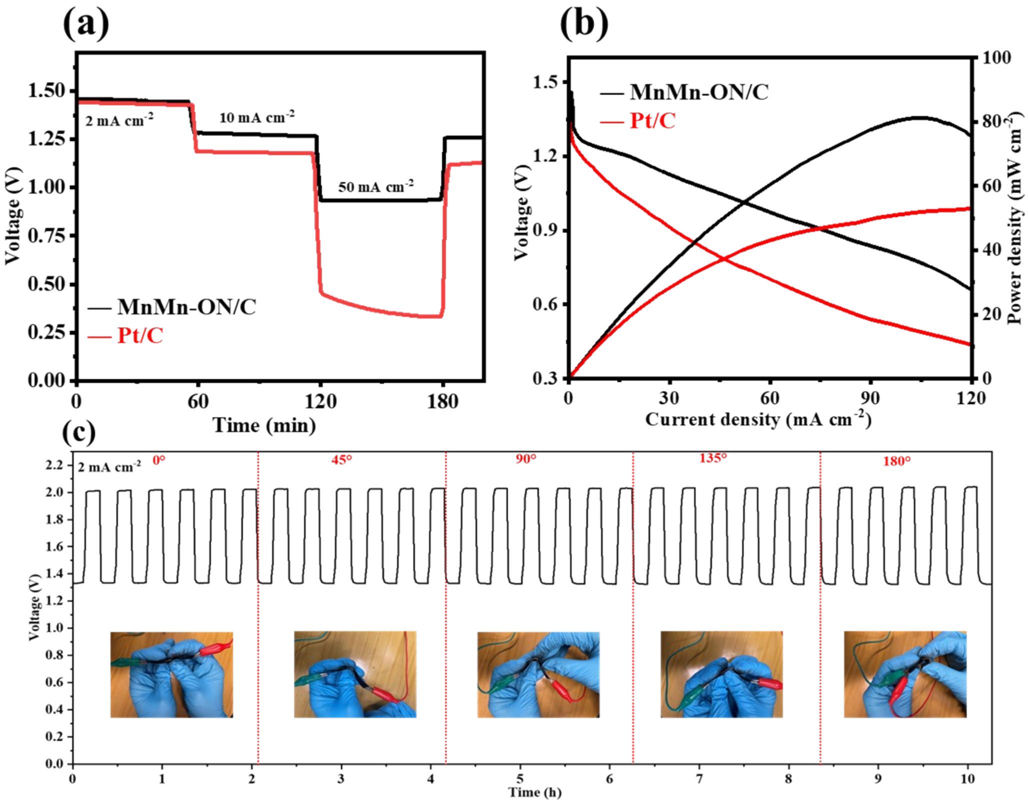

Fig. S15 (a) Discharge curves of the MnMn-ON/C catalyst applied in liquid zinc-air batteries at different current densities. (b) Comparison of the discharge polarization curves and power density of flexible zinc-air batteries. (c) Charge-discharge curves of flexible zinc-air batteries at different bending angles; the inset shows photographs at various bending angles.

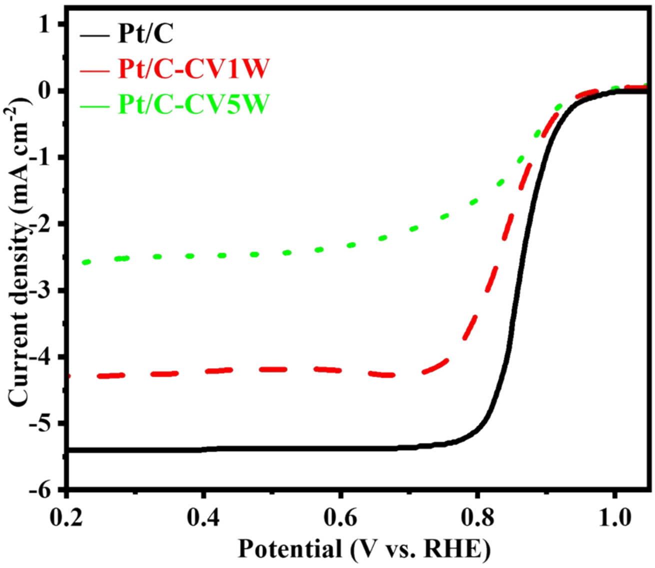

Fig. S16 Steady-state ORR polarization curves of Pt/C in O2-saturated 0.1 M KOH before and after 10,000 and 50,000 cycles.

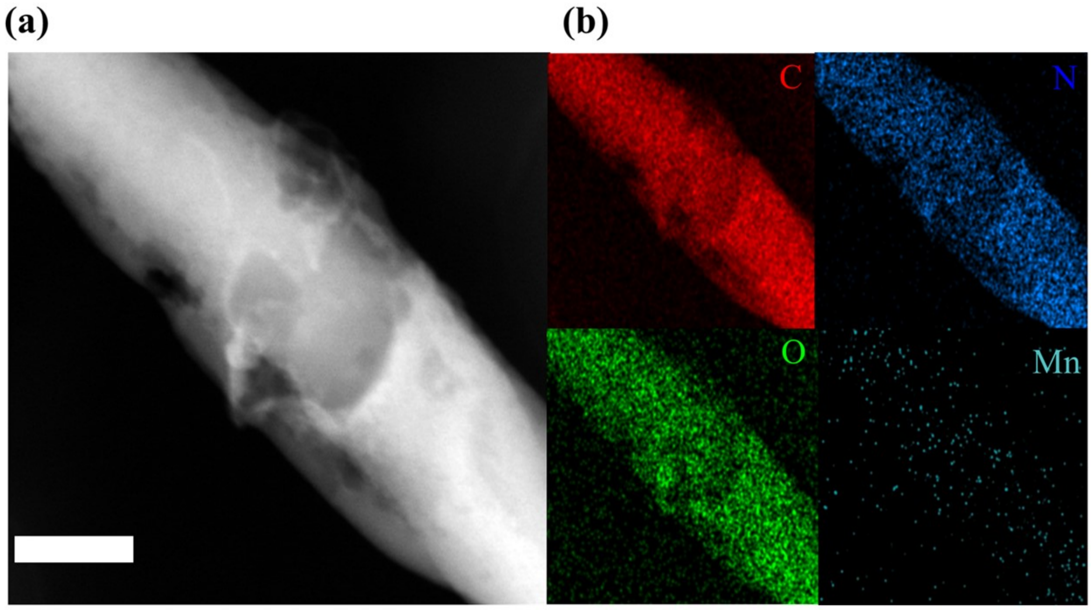

Fig. S17 (a) HAADF-STEM image of MnMn-N/C-CV5W (100 nm). (b) Elemental distribution map of the corresponding region.

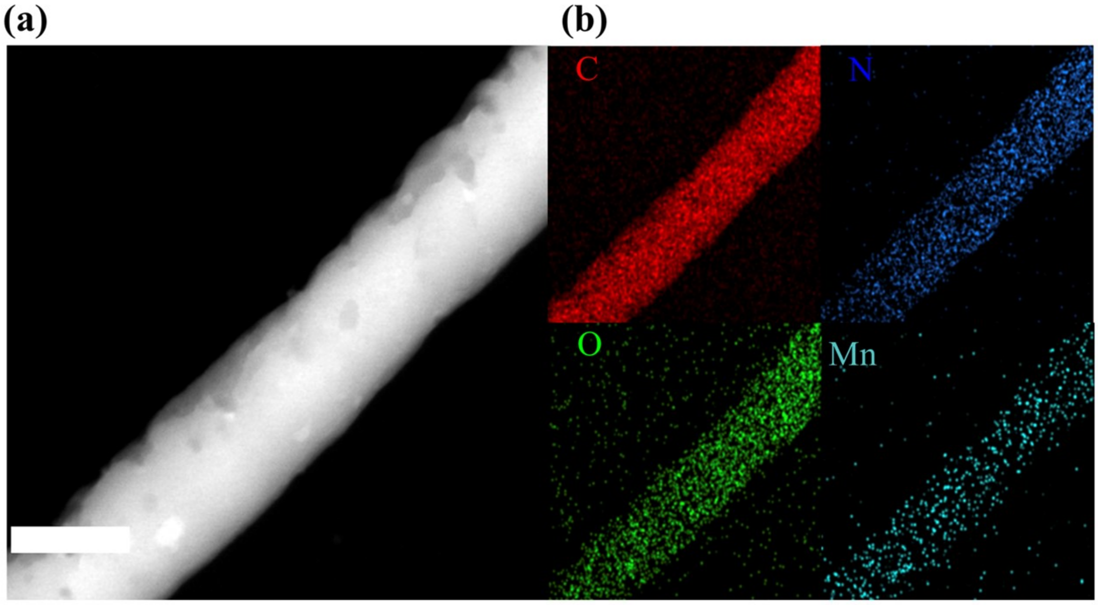

Fig. S18 (a) HAADF-STEM image of MnMn-ON/C-CV5W (100 nm). (b) Elemental distribution map of the corresponding region.

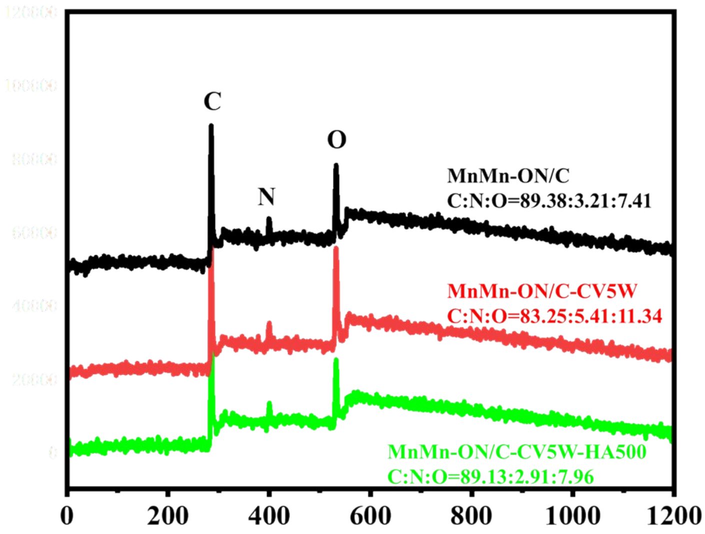

Fig. S19 XPS analysis of samples MnMn-ON/C, MnMn-ON/C-CV5W, and MnMn-ON/C-CV5W-HA500.

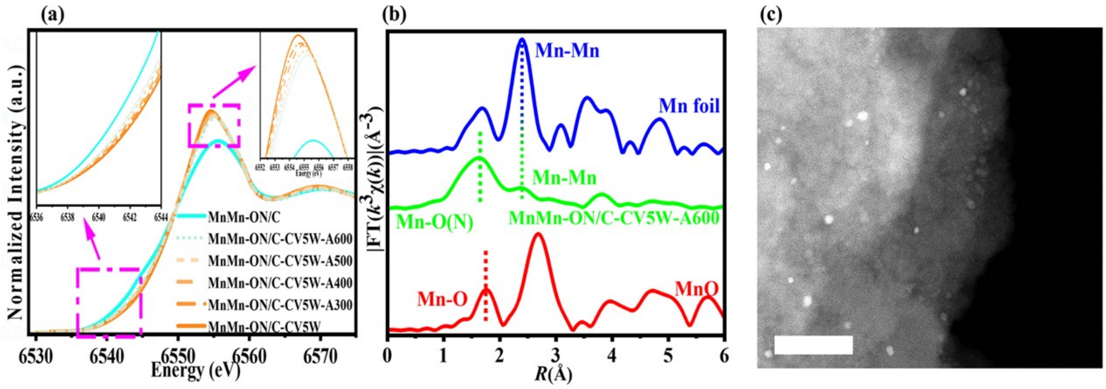

Fig. S20 (a) Mn K-edge XANES of MnMn-ON/C, MnMn-ON/C-CV5W-A600, MnMn-ON/C-CV5W-A500, MnMn-ON/C-CV5W-A400, MnMn-ON/C-CV5W-A300, and MnMn-ON/C-CV5W. (b) Fourier transform EXAFS spectra of MnMn-ON/C-CV5W-A600, Mn foil, and MnO. (c) TEM image of MnMn-ON/C-CV5W-A600 (50 nm).

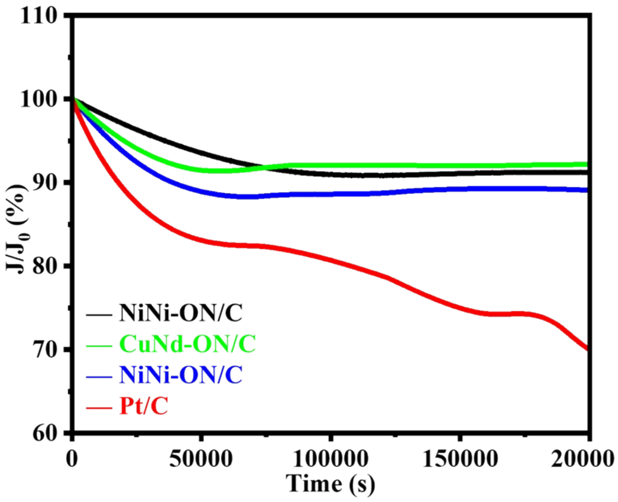

Fig. S21 Durability tests of NiNi-ON/C, CoCo-ON/C, CuCu-ON/C, and Pt/C.

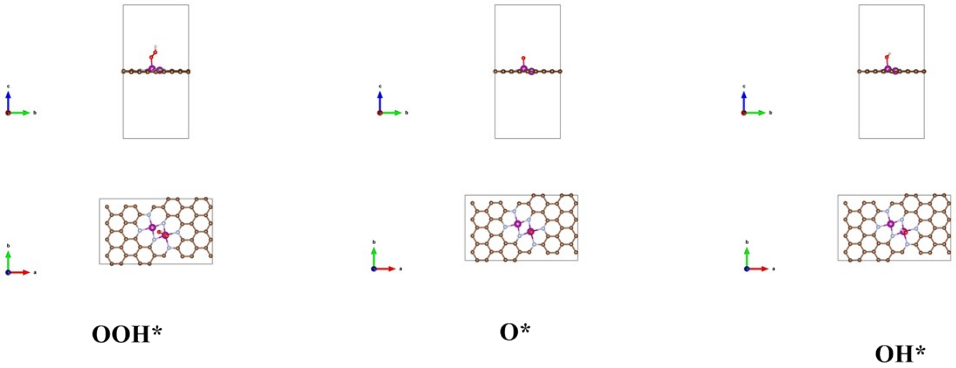

Fig. S22 Top and side views of three intermediate reaction steps for MnMn-N/C.

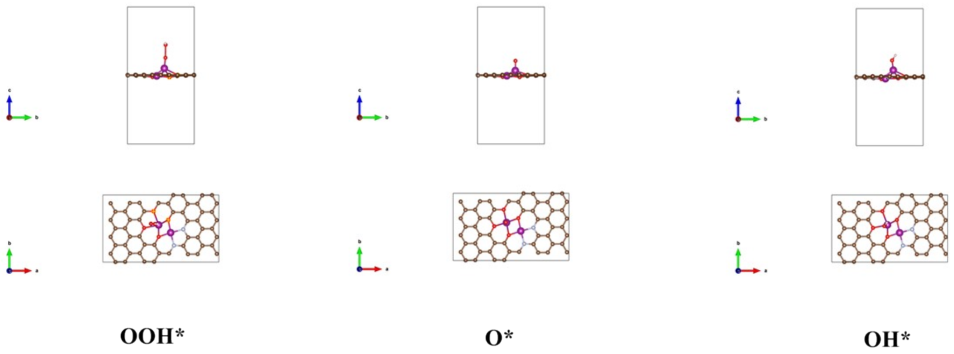

Fig. S23 Top and side views of three intermediate reaction steps for MnMn-ON/C.

Table S1. Shell paths are separated by scattering pair (e.g., Mn–N, Mn–O), but do not distinguish specific crystallographic sites. The reported coordination numbers (CN) are site averages across all Mn centers. Fitting of Mn species in MnMn-ON/C; fitting of Ni species in NiNi-ON/C; fitting of Co species in CoCo-ON/C; and fitting of Cu species in CuCu-ON/C. N represents the coordination number; σ^2 is the Debye-Waller factor characterizing disorder; R is the bond length between the central atom and the backscattering atom; ΔE0 is the inner potential correction value; and the R-factor is used to evaluate the goodness of fit.

Sample	Path	N	$\sigma^2(10^{-3}Å^2)$	R(Å)	ΔE_0(eV)	R factor
MnMn-N/C	Mn-N	4.0±0.1	4.38±0.3	1.80±0.11	1.35±0.22	0.016
	Mn-Mn	1.1±0.1	5.16±0.5	2.80±0.13		
MnMn-ON/C	Mn-N	1.1±0.1	4.47±0.4	1.77±0.12	1.45±0.52	0.018
	Mn-O	3.0±0.1	4.85±0.4	1.96±0.12		
	Mn-Mn	0.9±0.1	5.86±0.6	2.78±0.15		
NiNi-ON/C	Ni-N	1.0±0.1	3.52±0.5	1.75±0.13	2.51±0.21	0.015
	Ni-O	2.9±0.1	3.84±0.4	1.96±0.11		
	Ni-Ni	0.9±0.1	4.74±0.5	2.71±0.13		
CoCo-ON/C	Co-N	1.1±0.1	3.56±0.2	1.76±0.12	2.42±0.65	0.012
	Co-O	3.1±0.1	3.38±0.2	1.94±0.11		
	Co-Co	1.0±0.1	4.65±0.4	2.76±0.17		
CuCu-ON/C	Cu-N	0.9±0.1	4.72±0.5	1.77±0.11	1.83±0.41	0.014
	Cu-O	3.1±0.1	4.93±0.5	1.98±0.14		
	Cu-Cu	0.9±0.1	5.45±0.2	2.83±0.15		

Table S2. Comparison of the oxygen reduction reaction performance of MnMn-ON/C catalysts with other studies.

**Material**	**Electrolyte**	**$E_{1/2}$ (V *vs.* RHE)**	**J_L (mA cm^{-2})**	**Tafel slope (mV dec^{-1})**	**References**
MnMn-ON/C	0.1 M KOH	0.925	5.98	65.56	This work
Co-N-CTS	0.1 M KOH	0.86	5.63	94.5	[8]
FeMn-NCH	0.1 M KOH	0.86	6.12	74.6	[9]
FeMn/NC	0.1 M KOH	0.90	5.67	75.3	[10]
Co-COF@MOF	0.1 M KOH	0.86	6.2	39	[11]
Fe-O2N2/G	0.1 M KOH	0.86	5.9	90	[12]
FeACs/NPS−HC	0.1 M KOH	0.87	5.97	73.2	[13]
Mn–N2C2	0.1 M KOH	0.91	5.0	69.5	[14]
MnN_3O_1	0.1 M KOH	0.86	5.7	72	[15]
Fe&Mn/-N-C	0.1 M KOH	0.9	5.8	66.7	[16]
$Mn_{cluster}$/MnDAC-N-C	0.1 M KOH	0.91	4.7	79.9	[17]
FeMn(mlm)-N-C	0.1 M KOH	0.86	5.3	83	[18]
MnSAs/S–NC	0.1 M KOH	0.91	5.1	69	[19]

Table S3. Performance of MnMn-ON/C Catalysts in Zinc-Air Batteries Compared to Other Studies.

**Material**	**OCP (V)**	**Power density (mW cm^{-2})**	**Specific capacity (mAh g_{Zn}^{-1})**	**References**
MnMn-ON/C	1.55	209.1	817.7	This work
CoCo-BiSalphen@KB	1.512	205.7	816.3	[20]
FeCo/Se-CNT	1.54	173.4	745	[21]
NiCo2O4@FeNi LDH-4	1.40	130	810	[22]
NiFeOx/NP-C-800	1.40	82.5	688	[23]
FeCu-DSAs/NSC	1.468	180.66	817	[24]
FeCu-NC	1.47	91.2	795	[25]
R-CoMoO4/Co3O4@CC	1.43	120.3	654	[26]
PtSA-PtCo NCs/N-CNT-900	1.50	110.6	794.6	[27]
CoSAC@FePc	1.455	238.3	731.0	[28]
FeSA/AC@HNC	1.49	171.5	811.8	[29]
Fe/CoSx-SNC	1.452	156.6	784.4	[30]
FeCo-N3O3@C	1.43	143	787.2	[31]

Table S4. Determination of metal Mn content in several samples by ICP

Sample	MnMn-N/C	MnMn-N/C-CV1W	MnMn-N/C-CV5W	MnMn-ON/C	MnMn-ON/C-CV5W	MnMn-ON/C-CV5W-HA500	MnMn-ON/C-CV5W-A500	MnMn-ON/C-CV5W-A600
Content (wt%)	4.23%	3.59%	2.03%	4.20%	3.91%	3.89%	3.90%	3.89%

Table S5. The metal content (wt%) of the synthesized NiNi-ON/C, CoCo-ON/C, and CuCu-ON/C before and after durability testing was determined by ICP analysis.

Sample	Before durability testing	After durability testing
NiNi-ON/C	4.21%	3.99%
CoCo-ON/C	4.33%	3.93%
CuCu-ON/C	4.02%	3.81%

References

[1] Ma, Y.; Fan, H.; Wu, C.; Zhang, M.; Yu, J.; Song, L.; Li, K.; He, J. An Efficient Dual-Metal Single-Atom Catalyst for Bifunctional Catalysis in Zinc-Air Batteries. Carbon 2021, 185, 526–535. https://doi.org/10.1016/j.carbon.2021.09.044.

[2] Pu, T.; Ding, J.; Zhang, F.; Wang, K.; Cao, N.; Hensen, E. J. M.; Xie, P. Dual Atom Catalysts for Energy and Environmental Applications. Angew Chem Int Ed 2023, 62 (40). https://doi.org/10.1002/anie.202305964.

[3] Hafner, J. Ab-initioSimulations of Materials Using VASP: Density-functional Theory and Beyond. J Comput Chem 2008, 29 (13), 2044–2078. https://doi.org/10.1002/jcc.21057.

[4] Blöchl P E. Projector augmented-wave method[J]. Physical Review B, 1994, 50(24): 17953. https://link.aps.org/doi/10.1103/PhysRevB.50.17953

[5] Perdew, J. P., Burke, K. & Ernzerhof, M. Generalized gradient approximation made simple. Physical Review Letters, 1996, 77, 38653868.

[6] Momma, K.; Izumi, F. VESTA: A Three-Dimensional Visualization System for Electronic and Structural Analysis. J Appl Crystallogr 2008, 41 (3), 653–658. https://doi.org/10.1107/s0021889808012016.

[7] Wang, V.; Xu, N.; Liu, J.-C.; Tang, G.; Geng, W.-T. VASPKIT: A User-Friendly Interface Facilitating High-Throughput Computing and Analysis Using VASP Code. Computer Physics Communications 2021, 267, 108033. https://doi.org/10.1016/j.cpc.2021.108033.

[8] Shi, W.; Li, Z.; Gong, Z.; Liang, Z.; Liu, H.; Han, Y.-C.; Niu, H.; Song, B.; Chi, X.; Zhou, J.; Wang, H.; Xia, B. Y.; Yao, Y.; Tian, Z.-Q. Transient and General Synthesis of High-Density and Ultrasmall Nanoparticles on Two-Dimensional Porous Carbon via Coordinated Carbothermal Shock. Nature Communications, 2023, 14. https://doi.org/10.1038/s41467-023-38023-5.

[9] Jiang, Z.; Liu, X.; Liu, X.-Z.; Huang, S.; Liu, Y.; Yao, Z.-C.; Zhang, Y.; Zhang, Q.-H.; Gu, L.; Zheng, L.-R.; Li, L.; Zhang, J.; Fan, Y.; Tang, T.; Zhuang, Z.; Hu, J.-S. Interfacial Assembly of Binary Atomic Metal-Nx Sites for High-Performance Energy

Devices. Nature Communications, 2023, 14. https://doi.org/10.1038/s41467-023-37529-2.
[10] Xu, X.; Li, X.; Lu, W.; Sun, X.; Huang, H.; Cui, X.; Li, L.; Zou, X.; Zheng, W.; Zhao, X. Collective Effect in a Multicomponent Ensemble Combining Single Atoms and Nanoparticles for Efficient and Durable Oxygen Reduction. Angewandte Chemie International Edition, 2024, 63. https://doi.org/10.1002/anie.202400765.
[11] Yang, S.; Wei, Y.; Li, X.; Mao, J.; Mei, B.; Xu, Q.; Li, X.; Jiang, Z. Construction of High-Density Binuclear Site Catalysts from Double Framework Interfaces at the Cooling Stage. Angewandte Chemie International Edition, 2023, 62. https://doi.org/10.1002/anie.202313029.
[12] Li, Y.; Ding, Y.; Zhang, B.; Huang, Y.; Qi, H.; Das, P.; Zhang, L.; Wang, X.; Wu, Z.-S.; Bao, X. N,O Symmetric Double Coordination of an Unsaturated Fe Single-Atom Confined within a Graphene Framework for Extraordinarily Boosting Oxygen Reduction in Zn–Air Batteries. Energy & Environmental Science, 2023, 16, 2629–2636. https://doi.org/10.1039/d3ee00747b.
[13] Guo, X.; Shi, J.; Li, M.; Zhang, J.; Zheng, X.; Liu, Y.; Xi, B.; An, X.; Duan, Z.; Fan, Q.; Gao, F.; Xiong, S. Modulating Coordination of Iron Atom Clusters on N,P,S Triply-Doped Hollow Carbon Support towards Enhanced Electrocatalytic Oxygen Reduction. Angewandte Chemie, 2023, 135. https://doi.org/10.1002/ange.202314124.
[14] Shang, H.; Sun, W.; Sui, R.; Pei, J.; Zheng, L.; Dong, J.; Jiang, Z.; Zhou, D.; Zhuang, Z.; Chen, W.; Zhang, J.; Wang, D.; Li, Y. Engineering Isolated Mn–N2C2 Atomic Interface Sites for Efficient Bifunctional Oxygen Reduction and Evolution Reaction. Nano Lett. 2020, 20 (7), 5443–5450. https://doi.org/10.1021/acs.nanolett.0c01925.
[15] Y. Yang, K. Mao, S. Gao, H. Huang, G. Xia, Z. Lin, P. Jiang, C. Wang, H. Wang, Q. Chen, O-, N-Atoms-Coordinated Mn Cofactors within a Graphene Framework as Bioinspired Oxygen Reduction Reaction Electrocatalysts, Adv. Mater. 30 (2018) 1801732. https://doi.org/10.1002/adma.201801732.
[16] H. Cai, G. Zhang, X. Zhang, B. Chen, Z. Lu, H. Xu, R. Gao, C. Shi, Engineering the Local Coordination Environment and Density of FeN4 Sites by Mn Cooperation for

Electrocatalytic Oxygen Reduction, Small 18 (2022) 2200911. https://doi.org/10.1002/smll.202200911.

[17] Luo, G.; Zhu, E.; Shi, C.; Ren, Y.; Lin, Y.; Yang, X.; Xu, M. Regulating the Double-Site Mn2-N6 Electronic Structure by Manganese Clusters for Enhanced Oxygen Reduction. Applied Catalysis B: Environment and Energy 2024, 350, 123939. https://doi.org/10.1016/j.apcatb.2024.123939.

[18] S. Zhao, Z. Ma, Z. Wan, J. Li, X. Wang, Noble-Metal-Free FeMn-N-C catalyst for efficient oxygen reduction reaction in both alkaline and acidic media, Journal of Colloid and Interface Science 642 (2023) 800–809. https://doi.org/10.1016/j.jcis.2023.03.206.

[19] H. Shang, Z. Jiang, D. Zhou, J. Pei, Y. Wang, J. Dong, X. Zheng, J. Zhang, W. Chen, Engineering a metal–organic framework derived Mn–N4 –CxSy atomic interface for highly efficient oxygen reduction reaction, Chem. Sci. 11 (2020) 5994–5999. https://doi.org/10.1039/D0SC02343D.

[20] Zhang, Z.; Xing, Z.; Luo, X.; Cheng, C.; Liu, X. Densely Populated Macrocyclic Dicobalt Sites in Ladder Polymers for Low-Overpotential Oxygen Reduction Catalysis. Nature Communications, 2025, 16. https://doi.org/10.1038/s41467-025-56066-8.

[21] Zhang, H.; Zhao, M.; Liu, H.; Shi, S.; Wang, Z.; Zhang, B.; Song, L.; Shang, J.; Yang, Y.; Ma, C.; Zheng, L.; Han, Y.; Huang, W. Ultrastable FeCo Bifunctional Electrocatalyst on Se-Doped CNTs for Liquid and Flexible All-Solid-State Rechargeable Zn–Air Batteries. Nano Letters, 2021, 21, 2255–2264. https://doi.org/10.1021/acs.nanolett.1c00077.

[22] Wan, L.; Zhao, Z.; Chen, X.; Liu, P.-F.; Wang, P.; Xu, Z.; Lin, Y.; Wang, B. Controlled Synthesis of Bifunctional NiCo2O4@FeNi LDH Core–Shell Nanoarray Air Electrodes for Rechargeable Zinc–Air Batteries. ACS Sustainable Chemistry & Engineering, 2020, 8, 11079–11087. https://doi.org/10.1021/acssuschemeng.0c00442.

[23] Chen, X.; Wei, L.; Wang, Y.; Zhai, S.; Chen, Z.; Tan, S.; Zhou, Z.; Ng, A. K.; Liao, X.; Chen, Y. Milk Powder-Derived Bifunctional Oxygen Electrocatalysts for Rechargeable Zn-Air Battery. Energy Storage Materials, 2018, 11, 134–143. https://doi.org/10.1016/j.ensm.2017.10.011.

[24] Peng, J.; Hu, B.; Li, Z.; Zhong, X.; Shi, J.; Cui, S.; Wang, X.; Xu, B. Regulating

Atomic Fe/Cu Dual Sites with Unsymmetrical Fe-N6 and Cu-N1S2 Coordination for Promoting Bifunctional Oxygen Electrocatalysis in Advanced Zinc-Air Batteries. Energy Storage Materials, 2024, 68, 103342. https://doi.org/10.1016/j.ensm.2024.103342.
[25] Xu, M.; Zhang, L.; Liang, X.; Xiao, H.; Zhuang, H.; Zhang, F.; Zhang, T.; Han, P.; Dai, W.; Gao, F.; Zhang, J.; Zheng, L.; Gao, Q. Dual-Atom Fe(II,III)N2(M2-N)2Cu(I,II)N Moieties Anchored on Porous N-Doped Carbon Driving High-Efficiency Oxygen Reduction Reaction. Applied Catalysis B: Environment and Energy, 2024, 349, 123866. https://doi.org/10.1016/j.apcatb.2024.123866.
[26] Zhu, J.; Chen, J.; Li, X.; Luo, K.; Xiong, Z.; Zhou, Z.; Zhu, W.; Luo, Z.; Huang, J.; Li, Y. Steering Surface Reconstruction of Hybrid Metal Oxides for Efficient Oxygen Evolution Reaction in Water Splitting and Zinc-Air Batteries. Journal of Energy Chemistry, 2024, 92, 383–393. https://doi.org/10.1016/j.jechem.2024.01.020.
[27] Chen, W.; Zhu, X.; Wei, W.; Chen, H.; Dong, T.; Wang, R.; Liu, M.; (Ken) Ostrikov, K.; Peng, P.; Zang, S. Neighboring Platinum Atomic Sites Activate Platinum–Cobalt Nanoclusters as High-Performance ORR/OER/HER Electrocatalysts. Small, 2023, 19. https://doi.org/10.1002/smll.202304294.
[28] Xie, P.; Zhong, H.; Fang, L.; Lyu, Z.; Yu, W.; Li, T.; Lee, J.; Shin, H.; Beckman, S. P.; Lin, Y.; Ding, S.; Kim, I.; Li, J. Molecular Fe─N4 Moieties Coupled with Atomic Co─N4 Sites Toward Improved Oxygen Reduction Performance. Advanced Functional Materials, 2024, 34. https://doi.org/10.1002/adfm.202314554.
[29] Zhang, H.; Chen, H.; Feizpoor, S.; Li, L.; Zhang, X.; Xu, X.; Zhuang, Z.; Li, Z.; Hu, W.; Snyders, R.; Wang, D.; Wang, C. Tailoring Oxygen Reduction Reaction Kinetics of Fe−N−C Catalyst via Spin Manipulation for Efficient Zinc–Air Batteries. Advanced Materials, 2024, 36. https://doi.org/10.1002/adma.202400523.
[30] chen, Y.; Rong, J.; Wu, J.; Zhang, Y.; Ao, H.; Zheng, X.; Jing, C.; Yang, R.; Zhou, Z.; Li, Z. MOF-Derived S, N Co-Doped Porous Carbon Matrix with Single Fe Atoms and CoSx Nanoparticles Dual-Sites for Enhanced Oxygen Reduction. Chemical Engineering Journal, 2024, 502, 158080. https://doi.org/10.1016/j.cej.2024.158080.
[31] Tang, B.; Zhou, Y.; Ji, Q.; Zhuang, Z.; Zhang, L.; Wang, C.; Hu, H.; Wang, H.;

Mei, B.; Song, F.; Yang, S.; Weckhuysen, Bert. M.; Tan, H.; Wang, D.; Yan, W. A Janus Dual-Atom Catalyst for Electrocatalytic Oxygen Reduction and Evolution. Nature Synthesis, 2024, 3, 878–890. https://doi.org/10.1038/s44160-024-00545-1.
